# Non-Overlapping Progesterone Receptor Cistromes Contribute to Cell-Specific Transcriptional Outcomes

**DOI:** 10.1371/journal.pone.0035859

**Published:** 2012-04-24

**Authors:** Christine L. Clarke, J. Dinny Graham

**Affiliations:** Westmead Institute for Cancer Research, Sydney Medical School –Westmead, University of Sydney at the Westmead Millennium Institute, Westmead Hospital, Westmead, New South Wales, Australia; Baylor College of Medicine, United States of America

## Abstract

The transcriptional effects of the ovarian hormone progesterone are pleiotropic, and binding to DNA of the nuclear progesterone receptor (PR), a ligand-activated transcription factor, results in diverse outcomes in a range of target tissues. To determine whether distinct patterns of genomic interaction of PR contribute to the cell specificity of the PR transcriptome, we have compared the genomic binding sites for PR in breast cancer cells and immortalized normal breast cells. PR binding was correlated with transcriptional outcome in both cell lines, with 60% of progestin-regulated genes associated with one or more PR binding regions. There was a remarkably low overlap between the PR cistromes of the two cell lines, and a similarly low overlap in transcriptional targets. A conserved PR binding element was identified in PR binding regions from both cell lines, but there were distinct patterns of enrichment of known cofactor binding motifs, with FOXA1 sites over-represented in breast cancer cell binding regions and NF1 and AP-1 motifs uniquely enriched in the immortalized normal line. Downstream analyses suggested that differential cofactor availability may generate these distinct PR cistromes, indicating that cofactor levels may modulate PR specificity. Taken together these data suggest that cell-specificity of PR binding is determined by the coordinated effects of key binding cofactors.

## Introduction

Considerable effort has been applied over several decades to understanding the molecular mechanisms of progesterone signalling in target tissues such as the breast and endometrium. From comprehensive *in vitro* studies a detailed picture has emerged. Progesterone regulates transcription via its nuclear receptor (PR), which associates with specific target sites on chromatin. The consensus DNA sequence to which PR binds (progesterone response element (PRE)) consists of a six base pair inverted repeat sequence: RGNACAnnnTGTNCY [1], [2], [3]. DNA-bound PR recruits transcriptional coactivators and associated cofactors, which modify the local chromatin structure and facilitate transcriptional activation, resulting in activation or repression of PR target genes [4], [5], [6], [7]. In addition to coregulators and cofactors, which associate with the PR regulatory complex by protein-protein interaction, PR recruits chromatin remodelling factors, which modify local DNA architecture to enhance PR interaction and transcriptional activation [8]. Factors known to be involved in chromatin remodelling at progestin-regulated sites include the SWI/SNF chromatin remodelling complex [8], [9] and transcription factor NF1, which cooperates with PR for binding and activation of MMTV [10], [11]. For other nuclear receptors including estrogen (ER) and androgen receptor (AR), pioneer factors such as FOXA1, which interact with condensed chromatin, are required for nuclear receptor activation of transcriptional targets [12], [13], [14], [15], [16], [17]. In addition to direct interaction with DNA at PREs, PR has been reported to associate with target genes via tethering to other transcription factors, including AP-1, SP1 and Stat3 [18], [19], [20], [21], [22].

Although the critical determinants governing the transcriptional activity of PR have been described *in vitro*, the molecular basis for the strikingly pleiomorphic roles for this hormone *in vivo* are poorly understood. Progesterone is critical for normal reproductive tissue function [23] and in the uterus supports differentiation, and inhibits proliferation [24]. By contrast, in the breast progesterone is associated with increased proliferation, ductal side-branching and lobuloalveolar development [25]. Consistent with the distinct effects of progesterone in these two tissues, there are distinct transcriptional responses to progesterone in breast and endometrium [23], [26], [27], [28], [29], [30].

Exposure to exogenous progestins in hormone replacement therapy is associated with increased breast cancer risk [31], [32], [33], [34]. Interestingly, progestins regulate different transcriptomes in breast cancer cells compared with normal breast [35], so it is plausible that the effect of progestins on breast cancer risk may be mediated by altered specificity of progestin action in the cancerous breast. If altered cell-specificity of PR underlies the deleterious effect of progestins on breast cancer risk, the determinants of cell-specificity of progestin action require elucidation.

The DNA sequence of the response elements to which PR binds, the availability of transcriptional cofactors, and the chromatin architecture of the target cell are likely to have a combined effect on the specificity of the PR transcriptome. To determine the contribution of these variables to the cell-specificity of PR in normal breast and breast cancer cells, we used genome-wide PR chromatin immunoprecipitation, coupled with high-throughput sequencing to compare PR interaction on genomic DNA in two cell lines: T-47D cells and in MCF-10A immortalized normal breast cells stably expressing both PR isoforms. We report here on the discovery and characterisation of strikingly different PR cistromes in these two cell lines.

## Results

### Generation of genome-wide PR interaction profiles

PR genomic interactions were mapped in T-47D breast cancer cells and in the AB32 cell line: a stable PR expressing clone of the MCF-10A immortalized normal breast cell line. Cells were treated with the progestin ORG2058 (10 nM, 45 minutes), followed by PR-chromatin immunoprecipitation (ChIP) and Illumina sequencing. Sequences were aligned to the human genome and genomic regions enriched in the alignments were identified using the Bowtie [36] and ERANGE [37] software tools (false discovery rate 0.27%).

**Figure 1:**
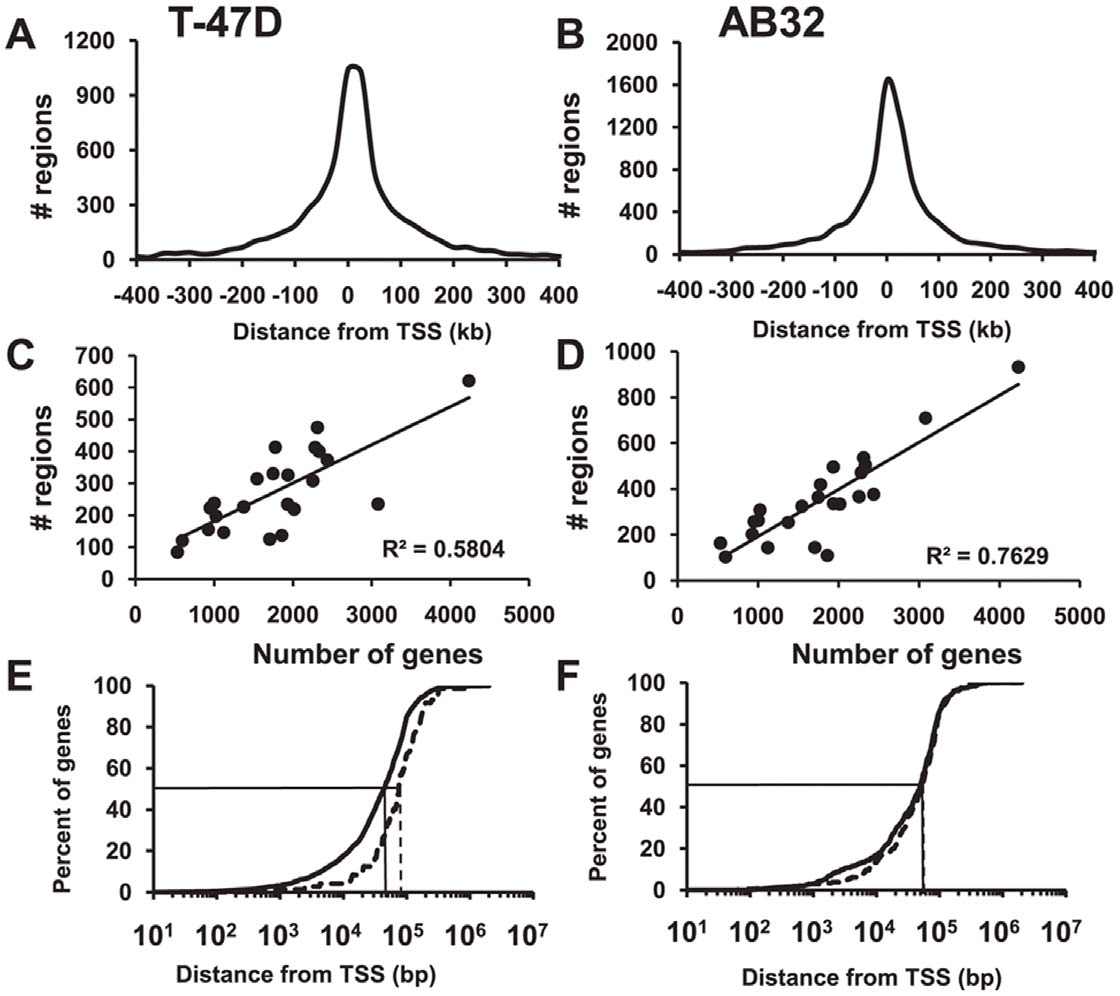
Genomic distribution of PR binding sites in T-47D and AB32 cells. Progestin-dependent PR bound DNA fragments identified by ChIP-seq were aligned to the genome using Bowtie and peaks of binding were identified using ERANGE, with 0.27% FDR. (A and B) Location of PR binding regions relative to transcription start sites of RefSeq genes in (A) T-47D and (B) AB32 cells was determined using CisGenome. (C and D) PR binding region distribution by chromosome, ranked by gene number in (C) T-47D and (D) AB32 cells. (E and F) Percentage of progestin regulated genes with PR binding regions within a given distance from the TSS in (E) T-47D and (F) AB32 cells. Solid line - binding regions associated with up-regulated genes, dashed line - binding regions associated with down regulated genes. The median distances of up- and down-regulated genes to nearest TSSs are indicated.

In T-47D cells, 6312 peaks of PR binding were identified and in AB32 cells 8117 binding regions were detected (Table 1). Most PR binding regions (88% in T-47D and 73% in AB32) were within 100 kb of the nearest gene, with 57% of binding regions in T-47D and 54% in AB32 within 50 kb. However, few binding regions (21% in T-47D and 20% in AB32) fell within 10 kb of a gene TSS (Table 1). The distribution of distances from the nearest gene transcription start site (TSS) for each of the PR binding regions in T-47D and AB32 cells is shown in Figure 1A and 1B, respectively.

**Table 1:** PR binding characteristics in T-47D and AB32 cells.

	T-47D		AB32	
	n	%	n	%
Regions total	6312	100	8117	100
Regions within 100 kb of a TSS	5548	87.9	5907	72.8
Regions within 50 kb of a TSS	3582	56.7	4385	54.0
Regions within 10 kb of a TSS	1308	20.7	1612	19.9
Regions near to regulated genes total	1239	19.6	1639	20.2
Regulated genes with regions within 100 kb of gene	559/950	59	749/1249	60
Regulated genes with region within 50 kb of TSS	414/559	74.1	517/749	69.0
Regulated genes with region within 10 kb of TSS	194/559	34.7	229/749	30.6
Regulated genes with region within 1 kb of TSS	46/559	8.2	56/749	7.5
Region to gene density overall	5548/7559 (0.73)	73.4	5907/7970 (0.74)	74.1
Region to regulated gene density	1239/559 (2.23)	223	1639/749 (2.19)	219
Region to up-regulated gene density	1169/509 (2.3)	230	1066/439 (2.43)	243
Region to down-regulated gene density	75/50 (1.5)	150.0	628/325 (1.93)	193
Regions near regulated genes that contain PREs	782	63.1	1010	61.6
Up-regulated genes associated with PR binding/total up-regulation	509/786	64.8	439/546	80.4
Down-regulated genes associated with PR binding/total down-regulation	50/98	51.0	325/621	52.3

PR binding regions were detected on all chromosomes and the number of binding sites per chromosome reflected chromosome size and number of genes, although some variability was observed. Linear regression analysis of binding regions against gene number per chromosome in T-47D (Figure 1C) and AB32 (Figure 1D) cells revealed a stronger correlation to gene number in the AB32 cells (R^2^ = 0.76 in AB32 compared to R^2^ = 0.58 in T-47D). Overall there was a correlation between numbers of binding regions per chromosome between the two cell lines (R^2^ = 0.66, Figure S1), however some exceptions were noted. Binding regions on chromosomes 2 and 8 were under-represented in the T-47D dataset compared to AB32, whereas regions on chromosome 11 were over-represented. The karyotype of T-47D cells [38] shows significant rearrangement and duplication compared to AB32 cells and this may partly explain the binding differences observed since T-47D cells contain 4 rather than 2 copies of chromosome 11. However, chromosome 2 is normal in T-47D cells, yet binding to regions on this chromosome were half as frequent as were detected in AB32 cells. Functional annotation of regulated genes on chromosome 2 that were bound by PR in AB32 revealed enrichment in genes involved in metabolism (Table S1), suggesting an altered or attenuated metabolic response to progestins in the cancer cell line.

### Relationship between PR genomic interaction and transcriptional response

Gene expression profiling conducted in parallel with ChIP-seq revealed that PR binding regions were concentrated around regulated genes. The density of PR binding regions per gene was higher for regulated genes (density of binding regions per regulated gene: 2.23 in T-47D cells, 2.19 in AB32 cells; Table 1) than the overall PR binding region density (0.73 per gene for all genes in T-47D cells, 0.74 in AB32 cells; Table 1). In addition, PR binding peaks were more likely to be within 50 kb of the gene transcription start site in regulated genes (74% and 69% of regulated genes in T-47D and AB32 cells), compared with the proportion of PR binding regions within 50 kb of all genes (57% of PR binding regions within 50 kb of TSSs in T-47D cells, 54% in AB32 cells, Table 1).

PR binding regions in T-47D cells were on average closer to up-regulated gene TSSs than regions near down-regulated genes. In T-47D cells the median distance of PR binding to up-regulated genes was 44 kb, whereas median distance to down-regulated genes was 75 kb (Figure 1E). This was reflected in a statistically significant overall difference in the cumulative frequency distributions of binding region distances to up-regulated and down-regulated genes in this cell line (Figure 1E, p = 0.001, Kolmogorov-Smirnov two-sample test). In contrast, no significant difference was seen in binding region distribution with respect to up- and down-regulated genes in AB32 cells (Figure 1F, p = 0.305).

In addition to PR binding regions being closer to up-regulated genes, there were more PR binding regions near up-regulated genes, with an average 2.3 binding regions per up-regulated gene compared with 1.5 per down-regulated gene in T-47D cells and 2.4 and 1.9 average regions per up- and down-regulated gene, respectively, in AB32 cells (Table 1). Moreover, a higher proportion of up-regulated genes (509/786 - 65% of up-regulated genes in T-47D and 439/546 - 80% of up-regulated genes in AB32) were associated with PR binding regions than down-regulated genes (50/98 - 51% of down-regulated genes in T-47D and 325/621 - 52% of down-regulated genes in AB32, Table 1).

### PR binding is associated with transcriptional regulation

**Figure 2:**
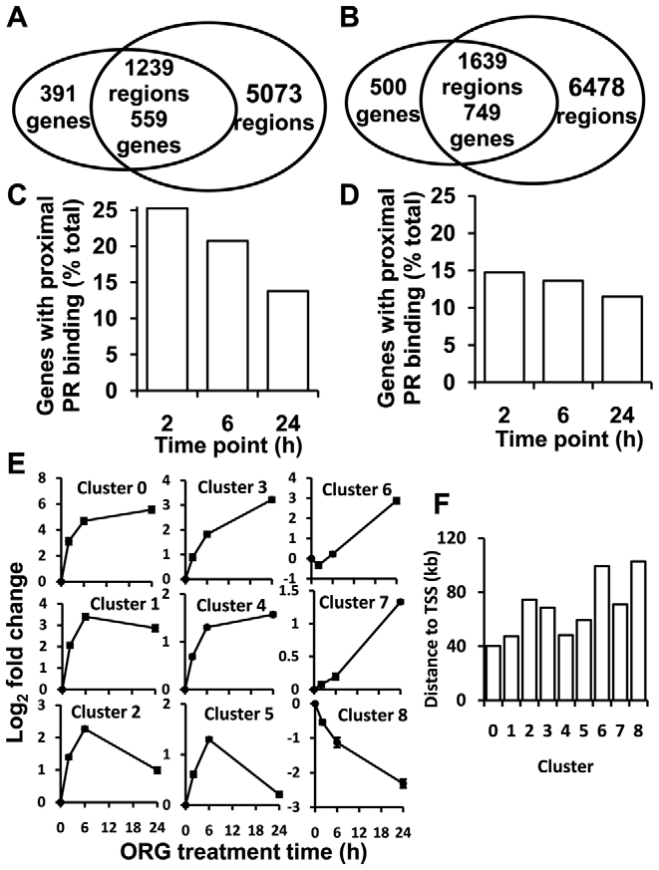
PR binding associated with progestin responsive genes. Gene expression profiles in T-47D and AB32 cells were determined at 2, 6 and 24 h after treatment with 10 nM ORG2058. Transcripts that were significantly differentially expressed at 2, 6 and/or 24 h, compared to untreated controls, were considered progestin regulated and were compared with the list of PR binding regions in the same cell line. (A and B) Overlap between PR binding regions and progestin regulated genes in (A) T-47D and (B) AB32 cells. (C and D) PR binding regions within 10 kb or in the 5′-UTR of genes regulated at 2, 6 and 24 h in (C) T-47D and (D) AB32 cells. (E) Genes with patterns of progestin regulation in T-47D cells that grouped together were identified by SOM cluster analysis using Gene Pattern. Patterns of regulation are plotted as the mean log fold change relative to the untreated control. Error bars represent the standard error of the mean. (F) Mean distance to TSS of PR binding regions associated with each SOM cluster shown in (E).

The majority of regulated genes in both cell lines (559/950 in T-47D (59%) and 749/1249 (60%) in AB32, Figure 2A and B, Table 1) had one or more PR binding region within 100 kb. There was a stronger association between PR binding and transcriptional regulation at earlier time points after ORG treatment, suggesting that genes that are directly regulated by PR are more likely to be detected early at the transcriptional level than those that are indirect targets (Figure S2). This relationship was strongest in T-47D cells, and in AB32 cells was true only for binding regions that were relatively near the TSS of regulated genes, as shown by the higher representation of promoter proximal PR binding regions (5′UTR and up to 10 kb upstream) at earlier times in both cell lines (Figure 2C and D). The overall distribution of PR binding regions with respect to intragenic and intergenic regions was similar in both cell lines (Figure S3): the greatest proportion of PR binding regions was observed upstream and in the 5′UTR of regulated genes, representing 43–45% of regions associated with regulated genes.

Self-organizing map (SOM) clustering of progestin-regulated transcripts associated with PR binding regions in T-47D cells (Figure 2E) and analysis of corresponding binding regions showed that PR binding regions were significantly closer to the TSSs of rapidly up-regulated genes, than to TSSs of down-regulated genes, or genes regulated at a later time point (Kruskal-Wallis one-way analysis of variance, p value<0.001, Figure 2F - compare clusters 0–5 with 6–8). Self-organizing map clustering of all progestin regulated transcripts revealed a pattern of regulation that was overall similar to that observed with the subset of genes associated with PR binding (Figure S4). However, a cluster of 26 transcripts was detected in the larger dataset representing transcripts that were decreased at all time points, but showed some recovery at 24 h. Although 12 transcripts in this cluster were also present in the set of transcripts associated with PR binding, 14 were found only in the full progestin regulated transcriptome, and represented transcripts that were regulated early but largely recovered by 24 h, suggesting that transcriptional silencing mediated by direct PR binding may be more sustained than indirect regulation.

In AB32 cells more transcripts were decreased than increased (Figure S5) and there was no significant difference in mean binding region to TSS distance observed between regulation clusters (Kruskal-Wallis p value = 0.209, not shown).

### Distinct patterns of PR binding between cell lines reflects divergent transcriptional response

**Figure 3:**
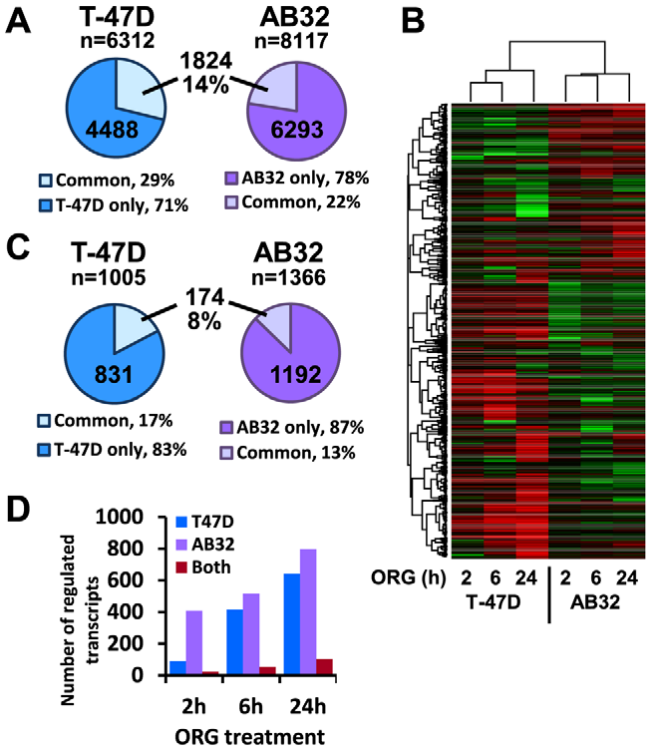
Differential PR binding and transcriptional regulation in T-47D and AB32. (A) PR binding regions that were common between T-47D and AB32 cells were identified using the IntersectBed function in Bed Tools. The number and percentage of regions that were unique to T-47D or AB32 cells or common to both cell lines are shown. (B) T-47D and AB32 cells were treated with 10 nM ORG2058 (ORG) or vehicle for 2, 6 and 24 h. Transcript expression was measured by whole genome microarray. Genes that were differentially expressed in ORG-treated samples relative to the untreated control at one or more time point in T-47D or AB32 cells were compared by unsupervised hierarchical cluster analysis. Red - increased expression, green - decreased expression relative to vehicle treated control. (C) Overlap between transcripts regulated by progestins in T-47D and AB32 cells at 2, 6 or 24 h. The numbers and percentage of transcripts that were uniquely regulated by progestins in T-47D or AB32 and regulation that was common to both cell lines are shown. (D) Numbers of progestin regulated transcripts in T-47D and AB32 at individual treatment times. The number of transcripts uniquely regulated in T-47D or AB32 cells or regulated in both at a specific time point is shown.

Of the 6312 regions bound by PR in T-47D and 8117 in AB32, just 1824 binding regions (14% of the combined total) were common to both cell lines, representing 29% of binding regions in T-47D and 22% of AB32 binding regions (Figure 3). The binding regions common to both cell lines were not more likely to be associated with regulated genes: of the 1824 binding regions found in both AB32 and T-47D, 431 (24%) were associated with progestin regulation in AB32 and 345 (19%) in T-47D - similar to the association of all binding regions with regulated genes shown in Table 1. Just 157 (9%) binding regions were associated with regulated genes in both cell lines (data not shown). Examples of binding peaks detected exclusively in one cell line or common to both are shown in Figure S6. Directed ChIP confirmed the differential patterns of PR binding to genes regulated in AB32, T-47D or both cell lines (Figure S7). Moreover, direct examination of the overlap between PR binding in T-47D and AB32 cells with another PR cistrome in T-47D cells [39] revealed a markedly higher overlap in binding regions between the two T-47D data sets than to the AB32 PR data set (Figure S8).

The lack of overlap in binding sites between the two cell lines was reflected in a similarly low overlap in transcriptional profiles at 2, 6 and 24 h of progestin treatment (Figure 3C–E). The small overlap in progestin targets in the two cell lines was similar at all time points examined (Figure 3E). This lack of overlap was confirmed in two additional cell lines, ZR-75-1 breast cancer cells and an additional PR+MCF-10A clone, AB9, which revealed a similarly low overlap of progestin response when compared directly with each other (Figure S9) or with the T-47D or AB32 cells.

### Conserved PRE identified in PR binding regions in T-47D and AB32 cells

**Figure 4:**
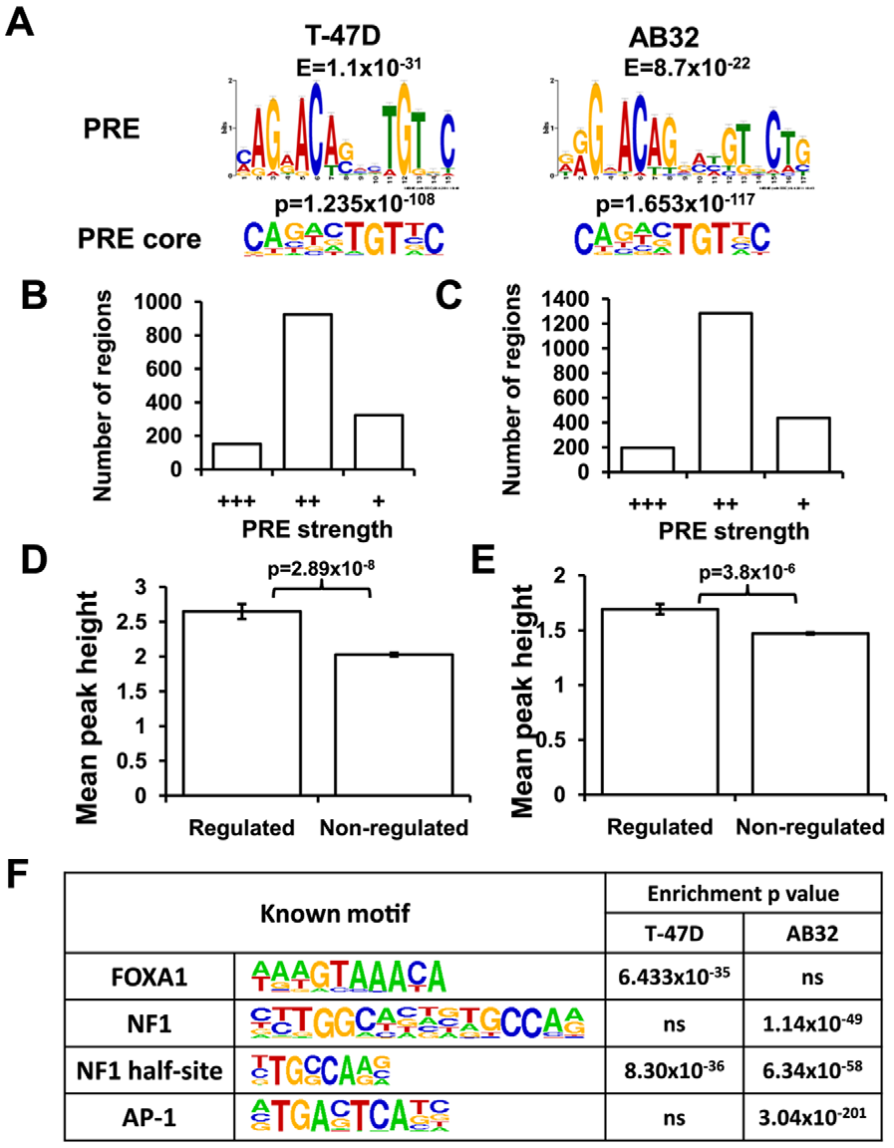
Motif enrichment in PR binding regions. (A) Full length and core PREs identified in binding regions in T-47D and AB32 cells. Regulation-associated PR binding region sequences were analysed for statistical enrichment of conserved sequence motifs using MEME-ChIP and Homer. Full and core PR binding elements were discovered in both cell lines. The full length PRE identified by MEME-ChIP and core element identified in Homer are shown. (B and C) PRE strength by p value in (B) T-47D and (C) AB32 cells. PREs were classified as strong (+++, p<1×10^−5^), moderate (++, p = 1×10^−5^ to 1×10^−3^) or weak/absent(+, p>1×10^−3^). (D and E) PR peak height in regulation and non-regulation associated PR binding regions in (D) T-47D and (E) AB32 cells. (F) Top transcription factor binding motif enrichment in T-47D and AB32 cells.

*De novo* motif enrichment analysis of PR binding regions associated with regulated genes identified highly significant enrichment of a conserved PR binding motif (Figure 4A) consistent with previously predicted progesterone response elements (PREs). Both the MEME-ChIP and Homer motif analysis tools identified a consensus PR binding motif consisting of a 6 bp inverted repeat sequence RGNACA separated by three non-specific bases, in agreement with classical biochemical studies of PR binding elements [2], [3] and similar to the element briefly described recently [39]. In addition, *de novo* analysis in Homer identified a highly enriched shorter element, representing the central core of the inverted repeat sequence (Figure 4A), suggesting that this more strongly conserved part of the PRE is most critical for PR binding. In T-47D cells 782/1239 PR binding regions (63%) associated with regulated genes contained one or more full-length or core PRE motifs (Figure S10A) and in 33% of binding regions this included at least one highly conserved full-length PRE. In AB32 cells 62% of regulation-associated PR binding regions contained PREs (Figure S10B). A substantial proportion of PR binding was likely to be mediated by a direct genomic interaction with these motifs, as there was a normal distribution of PREs about the centre of binding regions in both T-47D and AB32 cells (Figure S11, and one sample Kolmogorov-Smirnov test). A number of binding regions in both cell lines contained more than one PRE, although number of PREs was not correlated with peak height (Pearson regression R-squared = 0.0048), suggesting that PRE number was not correlated with binding strength. The position specific probability matrix for the full-length PREs defined by *de novo* motif mapping in the two cell lines was used to classify PRE strength in all PR binding regions. PRE strength did not predict a transcriptional outcome, since the same proportion of regulation associated and non-associated PR binding regions contained strong PREs (data not shown). Regulation-associated PR binding regions were grouped based on PRE p value (Figure 4B–C, strong (+++) - p<1×10^−5^, moderate (++) - p = 1×10^−5^–1×10^−3^, weak/absent (+) - p>1×10^−3^). By these criteria 77% regulation-associated binding regions in T-47D cells and 76% in AB32 contained one or more moderate or strong PRE (p<0.001). The majority of PR binding regions did not contain a strong PRE, suggesting broad flexibility in PR binding site selection and also implying that PR binding strength is not just determined by basic sequence and is likely influenced by secondary structure and other DNA binding PR cofactors. PR binding peak height was positively correlated with transcriptional outcome, suggesting that it is a measure of binding strength. In both T-47D and AB32 cells, the average peak height of binding regions that were within 50 kb of an up-regulated gene was significantly greater than those that were distant from any regulated gene (Figure 4D–E, unpaired t test, T-47D p = 2.89×10^−8^, AB32 p = 3.8×10^−6^). When PRE strength was compared directly with peak height, no correlation was observed, demonstrating that PRE quality alone does not determine PR binding strength (Figure S12). This finding was supported by directed ChIP validation of the top PR binding regions by peak height in T-47D and AB32 cells. Analysis of these regions revealed just two regions containing strong PREs in T-47D and three regions in AB32 cells. Most regions in the top ten contained moderate strength PREs, and binding of PR was confirmed in all but one binding region in each cell line (Table S2).

### Binding regions in T-47D and AB32 have distinct motif enrichment

We analysed the sequences up to 400 bp from each binding peak for the presence of other enriched motifs. Binding regions in T-47D cells were significantly enriched with motifs for the pioneer factor FOXA1 (Figure 4F), whereas there was no significant enrichment for this factor in PR binding regions from AB32 cells. FOXA1 binding motifs were identified in 548/1239 (44%) of regulation-associated PR binding regions in T-47D cells (Figure S10A). In binding regions in AB32 cells there was strong enrichment of binding sites for the AP-1 complex and for the DNA binding PR cofactor NF1. AP-1 binding motifs were present in 454/1639 (28%) and NF1 sites were identified in 380/1639 (23%) of regulation-associated PR binding regions in AB32 cells (Figure S10B). Relatively few binding regions in these cells (74/1639, 4.5%) contained binding motifs for both factors. There was no difference in the prevalence of any of the transcription factor motifs in binding regions near genes that were up- or down-regulated by progestin (t test, p>0.05). Moreover, separate motif analysis of up-regulation associated binding regions and of those associated with down-regulation did not reveal enrichment of different transcription factor motifs (not shown).

### The pioneer factor FOXA1 alters PR transcriptional response

**Figure 5:**
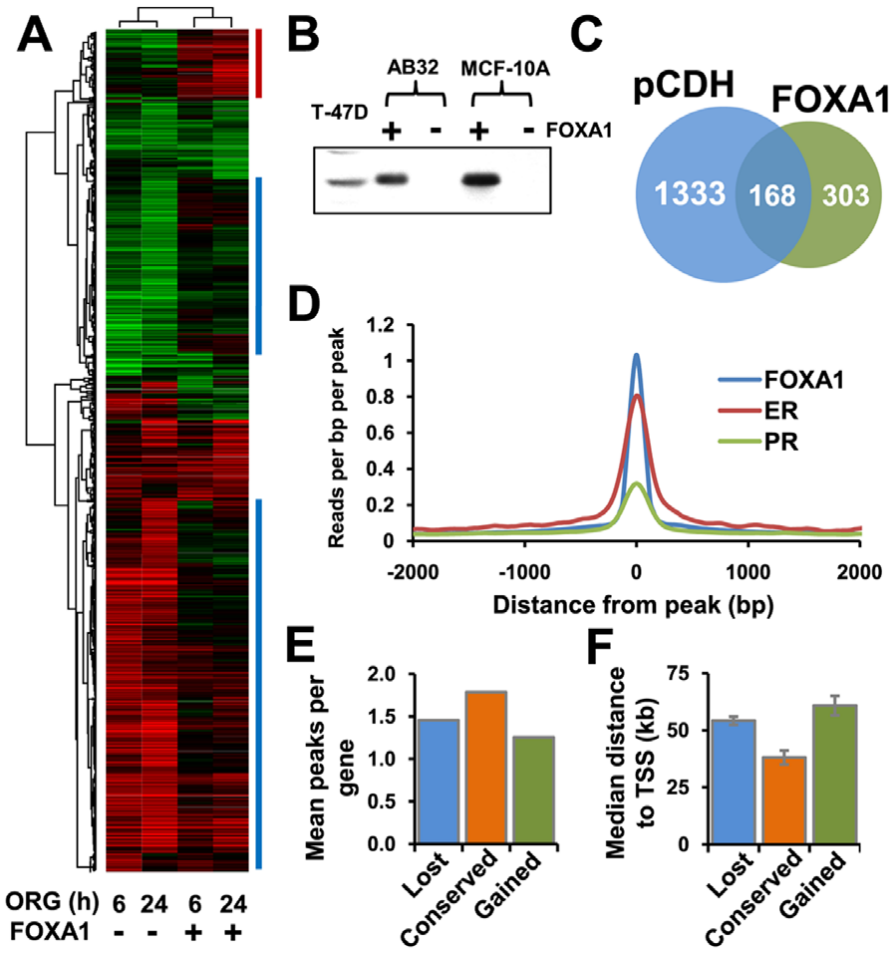
Introduction of FOXA1 into AB32 cells alters progestin response. (A) Unsupervised cluster analysis of transcriptional profiles in response to progestin in AB32 cells in the presence and absence of FOXA1. AB32 cells were transduced for 24 h with viral particles comprising the pCDH-FOXA1 construct or empty pCDH control. The cells were treated 6 and 24 h with 10 nM ORG2058 (ORG) or vehicle. Gene expression was measured by whole genome microarray. Genes that were differentially expressed at any time point in ORG-treated cells compared to control were analysed by unsupervised hierarchical cluster analysis. Red - increased log fold expression, green - decreased log fold expression. (B) FOXA1 protein expression in AB32 cells and parent MCF-10A cells before and after viral transduction, compared with endogenous expression in T-47D cells. (C) Numbers of progestin regulated transcripts in AB32 cells in the presence and absence of FOXA1. (D) Comparison of FOXA1 binding strength at PR, ER and FOXA1 binding sites. (E) Ratio of PR binding regions to regulated genes in AB32 cells for genes that lost, gained or retained progestin regulation with FOXA1 expression. (F) Distance from PR binding regions in AB32 to genes that lost, gained or retained progestin regulation with FOXA1 expression. Error bars represent standard error of the mean.

FOXA1 transcripts were abundantly expressed in T-47D and ZR-75-1 cells relative to AB32 and AB9 cells (Figure S13), suggesting that endogenous levels of FOXA1 may play a role in regulating the PR transcriptional response. Accordingly, AB32 cells lacking endogenous levels of FOXA1 were infected with lentiviral-delivered FOXA1 (Figure 5). This resulted in a profound alteration in the progestin-regulated transcriptome at 6 h and 24 h (Figure 5A). FOXA1 transduction resulted in progestin regulation of 303 transcripts that were not regulated in cells transduced only with the control pCDH virus (Figure 5C). Almost half of these targets (146/303, 48%) were detected as a distinct cassette of genes that clustered together (Figure 5A, red bar). Functional analysis revealed that genes that gained progestin regulation after FOXA1 expression were involved in blood vessel morphogenesis and regulation of cell motility (Table S3 and data not shown). These categories included genes such as transforming growth factor β3, CD44 and basic fibroblast growth factor, suggesting a broader developmental function. Surprisingly, a large proportion of transcripts that were regulated when FOXA1 was not present (1333 transcripts regulated at 6 h, 24 h or both, in absence of FOXA1, Figure 5C), lost regulation upon expression of the pioneer factor and were evident in multiple clusters (Figure 5A, blue bars). Functional analysis revealed a major impact of FOXA1 expression on genes involved in negative regulation of apoptosis: these had been increased by progestins in absence of FOXA1, but lost progestin responsiveness when FOXA1 was expressed (Table S3 and data not shown). Genes in this category that were decreased by progestin were unchanged by FOXA1 expression, suggesting that the net effect of FOXA1 was to promote apoptosis in response to progestin. The dampening effect of FOXA1 expression on progestin regulation suggested that the pioneer factor may play a dual role in PR action, similar to its role in androgen receptor signalling where it acts as an activator on a subset of androgen targets and a corepressor on others [40]. The progestin regulation of just 168 transcripts was unaffected by changed FOXA1 levels (Figure 5C). Functional analysis of these genes revealed progestin-mediated increases in genes involved in cell cycle progression, suggesting that the proliferative effects of progestin may not require FOXA1.

As FOXA1 appeared to have an effect on PR transcription distinct from that observed for ER, we compared the density of FOXA1 ChIP-seq interactions [41] around PR binding regions in T-47D cells, with those observed at FOXA1 or ER binding regions (Figure 5D). Binding of FOXA1 around ER binding regions was very high, confirming the absolute requirement for this factor in estrogen signalling. In contrast, although a peak of FOXA1 interaction was seen near PR binding regions, sequence enrichment was significantly lower (Figure 5D) suggesting that while FOXA1 may be involved in PR binding at some regions, this may represent a subset of binding events. This was supported by the finding that FOXA1 binding was much stronger at PR binding regions in which a FOXA1 motif had been predicted, than in regions where no motif was found, and was similar to the density of binding observed overall in ER binding regions (Figure S14). In order to test whether PR binding site numbers were different near genes that gained progestin-regulation upon FOXA1 expression, we compared the number of PR binding peaks in FOXA1 negative AB32 cells that were near to genes that lost, gained or retained progestin regulation when FOXA1 was expressed. Although there were slightly fewer PR binding regions near genes that gained regulation (Figure 5E), the difference was not significant. This suggested that the capacity of FOXA1 to influence PR binding and transcriptional regulation of target genes was not inherently related to PR binding site density; PR may form weak associations near to the “gained” subset of genes, but FOXA1 was required for the interaction to become productive. We also examined the level of enrichment of motifs for NF1 and AP-1 in PR binding regions associated with genes that lost, gained or conserved progestin regulation when FOXA1 was expressed and found no difference between the groups (not shown).

FOXA1 influences transcription factor activity via its DNA bending activity [42], [43], [44]. We speculated that PR binding regions that require FOXA1 to affect transcription may be further from the target gene than those that do not, and that binding of FOXA1 near those regions results in DNA bending, which brings the PR transcriptional complex closer to the target gene. Examination of the distance from PR binding regions to genes that gained regulation by FOXA1 revealed that this was the case and that this subset of regions was significantly further from the regulated gene than binding regions near genes regulated in the absence of FOXA1 (Figure 5F, p = 0.003, unpaired t test).

In summary, ChIP-seq profiling in two different cell lines has revealed remarkably distinct patterns of PR binding. These distinct cistromes are reflected in marked differences in transcriptional response to progestins. PR binding in the two cell lines is mediated by highly similar PREs, suggesting a similar mode of DNA interaction, but key differences in cofactor binding site enrichment, particularly FOXA1, suggest that the expression levels of these cofactors have potential to determine cell-specific binding and ligand response.

## Discussion

This first detailed genome-wide survey of PR genomic interaction has identified non-overlapping PR binding sites in immortalized normal and malignant breast cells; shown that PR interactions occurred distal to proximal promoters, supporting the view that PR effects are mediated over a longer distance than has previously been expected for direct *cis*-acting transcription factors; and demonstrated that transcriptional cofactors are important contributors to cell-specific PR activity.

### PR binding regions are distant from TSS

Most PR binding regions were located more than 10 kb from the TSS of regulated genes, with less than 35% of regulated genes in both cell lines having PR binding regions within 10 kb of the TSS, and less than 4% of regulated genes having binding regions within 1 kb of the TSS. In both breast cell types, binding was correlated with gene regulation, with most progestin-regulated genes having one or more PR binding regions within 50 kb, and genes increased by progestin being more likely to be associated with PR binding sites than genes that were decreased. These findings are consistent with observations for other nuclear receptors in comparable experimental systems. Reddy et al identified 4392 glucocorticoid receptor (GR) binding sites (2% FDR) by ChIP-seq in dexamethasone-treated A549 cells [45]. Welboren et al identified between 7713 and 10205 estrogen-dependent ER binding sites, depending on the peak-calling algorithm used, in MCF-7 cells [46]. Both ER and GR demonstrate a correlation between binding and gene regulation, and in line with the findings of this study, a relatively low proportion of promoter proximal binding is reported [14], [45], [46]. The stronger correlation between binding and transcriptional up-regulation than down-regulation has also been described for ER [14] and GR [45].

The number of PR binding sites discovered markedly exceeded the number of progestin-regulated transcriptional targets and many PR binding sites were not associated with active transcription, as only 20% of PR binding regions were associated with transcriptional regulation in each cell line. This has been found for other nuclear receptors [14], [17], [45], [46]. A number of potential explanations are proposed [47]. Some binding events may regulate transcription at a level below the detection threshold of genome-wide expression profiling. Moreover, a subset of binding sites may represent weaker associations or binding occurring in only a subset of cells such that transcriptional regulation does not occur at a significant level. Our data support this possibility, since PR binding peak signal strength was significantly higher near regulated genes compared to non-regulation associated binding regions. It has also been suggested that binding events that are not associated with transcriptional regulation may be at cell type specific sites requiring the co-operation of binding cofactors that are available only in a subset of contexts [47], [48], [49]. It must also be assumed that a proportion of binding regions represent non-specific interactions, although the finding that PREs are similarly prevalent in regulation-associated and non-associated binding regions would argue that non-specific interaction explains a small component of overall binding.

### PREs in PR binding regions

PR binding regions were significantly enriched for a binding element with a sequence consistent with previously described progesterone response elements [2], [3]. The relative conservation at specific base positions in the 15 bp palindromic response elements was variable, and was consistent with the pattern of conservation seen for GR [45], [49] and AR [50]. A shorter motif, representing the core highly conserved elements (CA/t nnn TGTnC, Figure 4A), was also detected, demonstrating the particular importance of these positions in the PR binding element. There was a high degree of variability of PRE sequences, as indicated by the consensus sequence allowing for marked variation at several positions, and many binding regions contained weak sequences that diverged considerably from the ideal. Moreover, a proportion of PR binding regions totally lacked a consensus PRE, raising the question of whether these were directly binding PR. To address this, we sought motif enrichment just in those regions, and did identify a PRE-like motif at a lower level of significance (not shown). This suggests that many binding regions lacking consensus PREs do contain sequences consistent with a PRE. A similar finding was reported for GR [45].

Although there was variability in the presence and strength of PREs identified in PR binding regions, this was not a determinant of whether a particular region was associated with transcriptional activity, as PRE strength was not correlated either with PR binding peak strength or with transcriptional outcome. This suggests that PRE strength is not the sole determinant of whether PR will interact with a particular binding region and regulate gene expression, and that other sequence features and the influence of DNA binding cofactors are likely to be important determinants. This is supported by the identification of FOXA1, AP-1 and NF1 as potential cell type-specific binding cofactors for PR in the two cell lines examined.

### The PR cistromes in T-47D and AB32 cells are non-overlapping

There was a relatively small overlap in PR binding regions in T-47D and AB32 cells. This was consistent with the observation that the transcriptional response to progestin was also non-overlapping between the two cell lines. Moreover, binding regions that were common to both cell lines were not more likely to be associated with a transcriptional outcome. Expression profiling in two additional cell lines, ZR-75-1 breast cancer cells and an independent PR positive MCF-10A clone (AB9), revealed a similarly low overlap in transcriptional regulation by progestins. It is important to note that the difference in PR cistromes observed in T-47D and AB32 cells may be contributed by lineage differences between these cells. MCF-10A cells (from which AB32 are derived) are considered to be myoepithelial in character, whereas T-47D cells have luminal characteristics. It is therefore possible that myoepithelial cells may respond differently to progesterone than luminal cells, even when PR is ectopically expressed, although confirmation of this possibility would require direct comparison of ectopic expression of PR into normal and malignant luminal or myoepithelial cells. Comparison of ER binding patterns in MCF-7 breast cancer cells and ER expressing U2OS osteosarcoma cells revealed a similarly low overlap in binding sites and transcriptomes [48]. In that study, differential promoter methylation was proposed to underlie this difference. However, global inhibition of DNA methylation in AB32 cells, while enhancing existing transcriptional targets, did not significantly alter the progestin-responsive transcriptome (data not shown). In support of our findings, Yin and colleagues have recently reported a similarly low overlap in PR genomic interactions in T-47D cells and uterine leiomyoma cells on exposure to the antagonist RU486 [51]. Cofactor motif enrichment was also found to be different between the two cell lines, however, some care should be taken in the interpretation of these data as no negative control or biological replicates for ChIP-seq were included in the study. Comparison of our T-47D and AB32 PR cistromes with a publicly available dataset revealed a markedly greater overlap between the two T-47D datasets (51% reported T-47D PR binding sites were also found in T-47D in our study) than with binding in AB32 cells (28%), supporting the validity of the observation of distinct binding patterns. The level of overlap in PR binding sites in T-47D cells from the two different studies was possibly influenced by a number of factors. The use of different ligands would influence the detection of PR binding sites. In the published study T-47D cells were treated with progesterone, which has a similar pharmacokinetic profile to ORG2058, but dissociates from PR more rapidly than the synthetic analogue. Moreover, the mobility of PR at genomic DNA has been shown to be ligand specific [52] and may differ when bound to ORG2058 compared to progesterone. Secondlly, PR binding was detected by different methods: in this study ChIP-seq was used, whereas the published data are derived from ChIP-chip. ChIP-seq surveys binding in an unbiased genome-wide fashion. ChIP-chip is dependent on the sequences present on the arrays used and can be affected by hybridization bias. A similar overlap was observed in ER binding sites detected in MCF-7 cells by ChIP-seq and ChIP-chip [46]. Lastly, the analysis methods used to generate the published data were likely different than used in our study. This information is not currently available for the published data.

### Role of chromatin structure and the pioneer factor FOXA1

Pioneer factors such as FOXA1 are able to bind to tightly packed heterochromatin, opening DNA structure to allow binding and regulation by nuclear receptors, including ER, GR and AR [12], [14], [15], [17], [40], [53]. The level of requirement for FOXA1 and the role that it plays in receptor signalling differs between the receptors. Expression of FOXA1 is critical for transcriptional activation by ER, although the specific gene targets may differ between cell lines. In a recent study, Hurtado and colleagues mapped ER and FOXA1 binding in three breast cancer cell lines, MCF-7, T-47D and ZR-75-1, and determined that positioning of the silencing factor CTCF was different between the three cell lines and defined which ER targets were transcriptionally enhanced by FOXA1 binding. In these cell lines FOXA1 was critical for ER action [15].

In contrast, FOXA1 appears to play a dual role in androgen signalling, where it promotes androgen responsiveness of some AR targets and acts as a repressor of others. This is supported by a recent study in LNCaP prostate cancer cells where depletion of FOXA1 caused significant remodelling of AR binding patterns and a marked increase in androgen regulated transcripts [40]. In this context FOXA1 is a critical determinant of binding site selection and acts both as a facilitator and a repressor of AR binding depending on the target site. Our data suggest that FOXA1 may play a similar role in PR signalling as with AR, since FOXA1 was not absolutely required for progestin response and over-expression of FOXA1 in AB32 cells, which lacked endogenous FOXA1, caused a marked decrease in the number of progestin-regulated genes in those cells. In T-47D cells where FOXA1 is abundantly expressed, binding motifs for the pioneer factor were statistically enriched in PR binding regions. The role of FOXA1 in PR signaling through regions containing FOXA1 motifs was supported by the finding that FOXA1 binding levels at these sites in T-47D cells was greater than interactions at PR binding regions that did not contain a predicted FOXA1 motif. However, a comparison of average FOXA1 binding around all PR binding regions in T-47D cells with those at ER interaction sites revealed significantly lower overall enrichment of FOXA1 binding near PR than ER, suggesting that FOXA1 is not required for all PR interactions. Taken together, our data suggest that FOXA1 may act as an enhancer of PR transcriptional activation of many of the targets identified in T-47D, whereas in AB32 cells the lack of FOXA1 expression allows binding of PR targets that may normally be repressed by FOXA1.

The overlap between progestin regulation in T-47D and FOXA1 transduced AB32 cells was low, suggesting that FOXA1 expression did not cause AB32 cells to become more like T-47D cells in their progestin response. This is consistent with our observation that FOXA1 may not be absolutely required for all PR binding events in T-47D cells. It also suggests that, although FOXA1 may affect PR binding, other cell specific factors or characteristics are important in determining PR binding, which may not be identifiable by ChIP. Both ER and AR have been shown to associate with histone modifying factors in a cell-type and promoter-specific fashion [54], [55], which are recruited to enhancers as part of a large coregulatory complex and would not be identifiable through motif analysis. The nature of the GR cistrome has been shown to be highly dependent on chromatin accessibility [56], which is also cell type specific. It is likely that the same factors influence PR binding in a cell type specific fashion.

### AP-1 and NF-1

Nuclear receptors, including PR, have been shown to associate with DNA independently of hormone response elements, by tethering to AP-1 [18], [19], [20]. In the case of ER, this mechanism was reported to mediate transcriptional repression of target transcripts by estrogen [14]. These findings suggest that AP-1 binding sites may be more common in binding regions that lack PREs and could be associated with down-regulated genes. AP-1 sites were present in 27% of regions that contained PREs and 29% of regions lacking PREs in AB32 binding regions. This proportion was higher overall than in T-47D cells where AP-1 site enrichment was not observed (12% regions with PREs and 10.7% regions lacking PREs contained AP-1 sites in T-47D), however it was not different between the two subsets of binding regions. There was also no evidence that AP1 sites were more prevalent in down-regulated than up-regulated genes (data not shown). These data suggest that, while AP-1 may co-operate with PR on a subset of binding sites in AB32 cells, its role in progesterone signaling may be more minor than for estrogen.

Binding of the transcriptional cofactor NF1 to DNA requires co-association by PR, and NF1 and PR have synergistic effects on gene expression [11], demonstrating the potential for co-expression of these transcription factors to result in enhanced transcriptional outcomes. In the mammary gland, the coordinated expression of NF1 isoforms is involved in controlling lactation and involution [57]. NF1 action in the mammary gland is context-specific, and is induced when mammary epithelial cells are maintained in contact with laminin-rich extracellular matrix [58]. The development-specific and context-specific actions of NF1 in the mammary gland suggest that its interplay with PR may be regulated by both NF1 and PR levels, and that these may be susceptible to modulation under physiological circumstances that include carcinogenesis. Enrichment of NF1 binding motifs in PR binding regions in AB32 cells, but not breast cancer cells, supports this view and suggests that NF1 is a cell type-restricted PR cofactor.

Our data suggest that a combination of chromatin remodelling cofactors is important for progesterone response in the breast and that the relative expression and coordinated action of these cofactors determines the PR cistrome. Progesterone has a diverse range of effects in normal and malignant target tissues and the results of this study demonstrate that the interplay between key cofactors and PR on the progesterone regulated cistrome contributes to context specificity of progesterone action, and may play a central role in aberrant progestin effects in breast cancer.

## Methods

### Cell culture

T-47D and ZR-75-1 breast cancer cell lines were obtained from the E.G. and G. Mason Research Institute (Worcester, MA). MCF-10A immortalized normal breast cells and HEK293T kidney cells were obtained from the American Type Culture Collection (atcc.org, Manassas, VA). T-47D and ZR-75-1 cells were maintained in RPMI1640 medium containing 10% fetal calf serum and 0.25 units/ml insulin. HEK293T were maintained in Dulbecco's Modified Eagle's Medium, supplemented with 10% fetal calf serum. The AB32 and AB9 cell lines were generated by co-introduction of PRA and PRB from viral vectors into the MCF-10A cell line and clonal selection using puromycin. Clones were characterised by dual immunofluorescent analysis and by western blotting for expression of PRA and PRB. A western blot comparing PR expression in AB32 and AB9 with PR levels in T-47D cells is shown in Figure S15. The cells were maintained in 1∶1 DMEM∶Hams-F12 medium supplemented with cholera toxin (0.1 µg/ml), insulin (0.28 iu/ml), hydrocortisone (0.5 µg/ml), epidermal growth factor (0.02 µg/µl), and 5% horse serum. The synthetic progestin ORG2058 was obtained from Amersham Biosciences (GE Healthcare, Rydalmere, Australia). TSA and 5AdC were obtained from Sigma-Aldrich (Castle Hill, Australia).

### Chromatin immunoprecipitation

Cells were cultured to 80% confluency in 15 cm tissue culture dishes, then treated for 45 minutes with 10 nM ORG or vehicle. Chromatin was subsequently cross-linked by the addition of formaldehyde to the culture medium to a final concentration of 1% and incubation for 10 minutes at 37°C. Media were immediately removed and the cells were washed with cold phosphate buffered saline and harvested by scraping. Cells were collected by centrifugation and pellets were lysed 10 minutes at 4°C in SDS buffer (1% SDS; 10 mM EDTA; 50 mM Tris-HCl, pH 8). The lysates were sonicated at 4°C with a Branson 450 sonicator, using seven one minute bursts at 40% amplitude and 60% duty, each separated by a rest of at least one minute. Lysates were centrifuged at 13,000× g at 4°C for 15 minutes to remove debris. Genomic DNA was isolated from an aliquot of lysate and checked by gel electrophoresis to confirm that sonication had resulted in fragmented DNA with an average size of 200 to 400 bp. Supernatants were diluted 1∶10 with IP buffer (0.5% NP-40; 50 mM Tris, pH 8; 120 mM NaCl; 0.5 mM PMSF; Complete protease inhibitor cocktail, Roche, Ryde, Australia) and pre-cleared using washed Dynabeads M-280 sheep anti-mouse IgG magnetic beads (Invitrogen, Mulgrave, Australia), with gentle rotation at 4°C for at least 2 h. Genomic DNA fragments that were bound to PR were isolated by rotation overnight at 4°C with in-house hPRa6 and hPRa7 mouse monoclonal anti-PR antibodies [59] and fresh sheep anti-mouse IgG magnetic beads (40 ul per 1.4 ml diluted lysate). Beads were washed sequentially with IP buffer, high salt wash buffer (0.5% NP-40, 50 mM Tris, pH 8, 500 mM NaCl, 0.5 mM PMSF), lithium wash buffer (250 mM LiCl, 0.5% NP-40, 1% sodium deoxycholate, 1 mM EDTA, pH 8, 10 mM Tris-HCl, pH 8) and TE (10 mM Tris, pH 8, 1 mM EDTA). Isolated DNA fragments were eluted twice for 15 minutes at room temperature using elution buffer (1% SDS, 0.1 M NaHCO3). Cross-links were reversed by addition of NaCl to 0.25 M and heating to 65°C for at least 6 h. DNA fragments were purified using Qiagen PCR purification columns (Qiagen, Doncaster, Australia). DNA fragments isolated by PR chromatin immunoprecipitation from ORG-treated cells were sequenced on an Illumina GA-IIx sequencer at the Ramaciotti Centre for Gene Function Analysis (University of New South Wales, Australia) and GeneWorks (Hindmarsh, Australia). Input DNA isolated from the pre-cleared ORG-treated samples were sequenced as a baseline control.

### Analysis

Sequences were aligned to repeat masked human genome hg18 (NCBI build 36) using Bowtie 0.12.0.1 [36]. Up to 2 mismatches were allowed in the aligned sequences. Multiple alignments were permitted up to a multiplicity of 10, but only the best ranked alignment was reported. This strategy resulted in alignment of 42 to 48% of reads. Results in T-47D represent the combined outcome of three independent biological replicates and two replicate input controls. AB32 results are from two independent ChIP and input control samples each. All sequences were at 36 bp read length except for one ChIP and one matched input control sample from T-47D cells. These samples were processed with a 64 bp read length, but were trimmed to 36 bp during data processing to avoid bias in the downstream analysis. Enriched regions of PR binding were determined using the ERANGE open source software tool [37]. Peak shift was determined to be 70 bp using the -shiftLearn function in findall.py. The peak threshold was set at four-fold background as determined from the control input DNA sequence. The minimum number of reads (RPM) within a region was set to 10. Multireads were weighted at a value of 1/multiplicity. Peaks were called with false discovery rate 0.27%. Regions of PR binding were annotated with respect to neighbouring genes using CisGenome v1.1 [60] and Homer [61]. Known and de novo enriched binding motifs were identified using Homer and the MEME suite of motif analysis tools, version 4 [62], [63]. Significance of enrichment of binding motifs discovered in MEME was determined using a Fisher Exact Test. The E-value for enrichment represents the p-value multiplied by the number of sequences tested. Motif enrichment was scored in Homer using a cumulative hypergeometric distribution analysis comparing binding region sequences with a matched genomic background [61]. The FIMO program in MEME was used to classify full-length PRE occurrences in PR binding regions in AB32 and T-47D cells, using the position specific probability matrices discovered by MEME in the two cell lines. Sequences with a p value<0.01 for similarity to the consensus PRE were reported and p values ranged from 0.01 to 1×10^−10^. A lower p value signified greater sequence conservation compared to the consensus PRE and for the purposes of comparisons, a p value<1×10^−5^ was considered to represent a strong PRE. For comparison of PR genomic interaction in T-47D cells with published ER and FOXA1 interactomes [41], sequence tag libraries were generated from all three data sets in Homer and binding peaks were identified using the same parameters for each data set. Average FOXA1 tag density was then determined at PR, ER and FOXA1 peaks using the peak annotation function in Homer. All raw data generated by ChIP-seq and gene expression profiling have been deposited on the Gene Expression Omnibus (www.ncbi.nlm.nih.gov/geo/) and can be accessed through GEO accession number GSE31130. Gene expression data conform to MIAME guidelines.

### Real-time PCR

Directed ChIP was performed using the same protocol as described for ChIP-seq. Target templates were quantitated using Platinum Sybr Green reagents (Invitrogen) in a RotorGene 6000 real-time PCR machine. Directed ChIP was carried out as described above and purified gDNA fragments were diluted 2 to 5-fold prior to quantitation by real-time qPCR. Primer sequences used for specific target validation were: ACSL1 - fwd 5′-TGC AAA GAG CAA GAC AGA AAA G-3′, rev - 5′-GCG GTC ATA GAG ACA CAA TTC C-3′, DHRS9 - fwd 5′-GGC TGT CTG AGT GAA TCT GTA GTG-3′, rev - 5′-AGT TAC ATT TGC CCT TGA TTC C-3′, FLRT3 - fwd 5′-GGA GAA ACA GAC TTT ACC TGA CC-3′, rev - 5′-TGT TGC AGT CAA GGA GAC AGA G-3′, NOTCH 2 - fwd 5′-GCC TGT TCC TAT TAA GTG TCC TG-3′, rev - 5′-GGC TGT AAA GTT ATT TGC TAG ATT G-3′, PACSIN1 - fwd 5′-AAC GTC CTC TTC CTG CTC TTG-3′, rev - 5′-GAG CTT TGA TGT AGA CGG AAT-3′ G, PDK4- fwd 5′-CG AGC AGC AAT AAC TTT CC-3′, rev - 5′-ACG CAA GAA CAC AGT GAG TAG C-3′.

### Lentiviral transduction

The FOXA1 cDNA was obtained from the PlasmID Dana Faber/Harvard Cancer Center DNA Resource Core (Boston, MA). The open reading frame was amplified by high fidelity PCR and transferred into the multiple cloning site of pCDH-CMV-MCS-EF1-copGFP. Integrity of the insert was confirmed by sequencing. Lentiviral particles were generated by cotransfecting the pCDH-FOXA1-GFP vector and lentiviral packaging constructs into HEK293T cells and allowing virus to accumulate in the medium for 48 h. Viral titre was estimated over a dilution series in AB32 cells, using a Facs Calibur flow cytometer to estimate GFP positivity. AB32 cells were infected at a level predicted to give a 70% infection rate and incubated for 24 h at 37°C to allow expression of FOXA1, followed by treatment for 0, 6 and 24 h with 10 nM ORG. Matched control samples infected with the parent pCDH-CMV-MCS-EF1-copGFP virus were included at each time point.

### Gene expression microarray

Total RNA was isolated using RNAqueous purification columns (Invitrogen). Total RNA (500 ng) was amplified and biotin labelled using Illumina TotalPrep reagents (Invitrogen). The amplified samples (750 ng) were hybridized to human whole genome HT-12 gene expression bead arrays using the recommended Illumina reagents and following the manufacturer's protocol. Raw data were analysed using Genome Studio software (Illumina). After background subtraction data and cubic spline normalization, differential expression p values were determined using the Illumina custom model of Genome Studio. Data were further analysed using Microsoft Excel and SPSS statistical software. Hierarchical clustering and self organizing map clustering were performed using GenePattern [64].

### Protein extract preparation and immunoblotting

Cells to be analysed by protein immunoblot were harvested by trypsinization, washed with cold phosphate buffered saline solution and collected into a cell pellet by centrifugation. Whole cell extracts were prepared by lysis of cells in RIPA buffer (10 mM NaPO_4_ (pH 7.0), 150 mM NaCl, 2 mM EDTA, 1% sodium deoxycholate, 1% NP-40, 0.1% β-mercaptoethanol) containing 10 mM NaMoO_4_, 1% aprotinin, Complete protease inhibitor (Roche, Castle Hill, Australia) and 0.5 mM phenylmethylsulfonylfluoride, and rotation 15 min at 4°C. Insoluble debris was removed by centrifugation at 14,000× g, 15 min at 4°C. Protein concentration was estimated using Bradford dye reagent (Bio-Rad, Regents Park, Australia). Proteins were fractionated by electrophoresis through denaturing 7.5% polyacrylamide-SDS gel and transferred to nitrocellulose membrane as described previously [65]. For detection of FOXA1 expression T-47D whole cell extract was loaded at 100 µg per lane and transduced cell extracts at 10 µg per lane. FOXA1 was detected using a goat anti-human FOXA1 polyclonal antibody (Abcam ab5089, Sapphire Biosciences, Waterloo, Australia) at 1∶800 dilution, and rabbit anti-goat horseradish peroxidase conjugated secondary antibody (Dako Cytomation, Kingsgrove, Australia). For detection of PR protein expression, whole cell extracts were loaded as indicated. PR was detected using hPRa6 and hPRa7 in-house mouse monoclonal antibodies (1∶100 each) and goat anti-mouse horseradish peroxidase conjugated secondary antibody (Dako). Protein bands were visualized by chemiluminescent reaction using ECL reagents (Quantum Scientific, Murrarie, Australia) and exposure to film or imaging using a Kodak Image Station (Carestream Health, Richmond, Australia).

## Supporting Information

Figure S1 **PR binding region to chromosome distribution in T-47D and AB32 cells.** Total numbers of PR binding region per chromosome were compared by linear regression between T-47D and AB32 datasets. Line of fit and 95% confidence intervals are shown. Labels represent chromosome number.
(PDF)

Figure S2 **Relationship between PR binding and time of progestin regulation.** The proportion of progestin regulated genes at 2, 6 or 24 h after treatment, which were associated with one or more PR binding regions was determined in (A) T-47D and (B) AB32 cells.
(PDF)

Figure S3 **Location of PR binding regions.** The distribution of all PR binding regions, with respect to the nearest gene, was determined using CisGenome v1.1 in (A) T-47D and (B) AB32 cells.
(PDF)

Figure S4 **Patterns of transcriptional regulation in T-47D cells.** Transcripts that were significantly differently expressed in 10 nM ORG2058 treated cells relative to vehicle at 2, 6 and 24 h after treatment were identified by gene expression profiling on Illumina HT-12 whole genome array. Self-organising map clustering was performed for all progestin regulated genes, using Gene Pattern.
(PDF)

Figure S5 **Patterns of transcriptional regulation in AB32 cells.** Progestin regulated transcripts were identified in AB32 cells at 2, 6 and 24 h treatment with 10 nM ORG2058 by gene expression profiling. Patterns of transcriptional regulation over the 24 h time course were identified by self-organising map clustering using Gene Pattern.
(PDF)

Figure S6 **PR binding regions in T-47D and AB32 cells.** Examples of PR binding regions that were unique to T-47D or AB32 cells or common to both lines are shown as custom tracks displayed in the UCSC genome browser.
(PDF)

Figure S7 **Validation of cell type-specific PR binding regions identified in ChIP-seq.** PR binding regions identified in (A) T-47D, (B) AB32 or (C) both cell lines by ChIP-seq were validated by directed PR-ChIP, using binding region-specific primers and quantitative real-time PCR. Regions bound near ACSL1 and PACSIN1, which were regulated in T-47D but not AB32 produced marked enrichment of bound fragments in T-47D cells and not AB32. The converse was true with PR target regions identified in AB32 but not T-47D. FLRT3 and DHRS9, which are both transcriptional targets only in AB32, were strongly bound by PR in AB32 but showed a weak association in T-47D cells. PDK4 and Notch 2, which are progestin regulated in both cell lines, were bound by PR in both although the association was stronger in T-47D (85-fold vs42-fold binding enrichment of PDK-4 and 300-fold vs37-fold enrichment of Notch 2 binding in T-47D vs AB32).
(PDF)

Figure S8 **Overlap of PR binding regions in ORG2058-treated T-47D and AB32 with binding in T-47D after progesterone (P4) treatment.** Our data are compared with progesterone-liganded PR binding in T-47D summarized in Tang et al [39] and available at http://cistrome.dfci.harvard.edu/NR_Cistrome/.
(PDF)

Figure S9 **Progestin regulation of gene expression in additional breast cell lines.** ZR-75-1 breast cancer cells and AB9 PR-positive transformed normal breast cells were treated for 2, 6 or 24 h with 10 nM ORG2058 or vehicle, then harvested and total RNA was isolated. Gene expression levels were estimated by Illumina HT-12 microarray. Data were analysed using Genome Studio software. Transcripts with levels that were significantly different in ORG compared to vehicle-treated cells (diff p value<0.01) and had a fold change of 1.5 or more were considered progestin regulated. (A) Numbers of progestin regulated transcripts in ZR-75-1 or AB9 cells or both. (B) Unsupervised average linkage hierarchical cluster analysis of arrays (Pearson correlation) and gene expression fold change (uncentred correlation) was performed on the subset of transcripts that were progestin regulated in one or both cell lines, using Gene Pattern. Red - increased expression, green - decreased expression with ORG, relative to vehicle.
(PDF)

Figure S10 **PRE and cofactor motif enrichment in regulation-associated binding sites in T-47D and AB32 cells.** The relative proportions of regulation-associated PR binding regions containing PREs with or without one or more of the top enriched transcriptional cofactor binding motifs are shown. (A) T-47D and (B) AB32 motif distribution.
(PDF)

Figure S11 **Distribution of PRE position in PR binding regions in T-47D and AB32 cells.** The positions of PRE motifs in PR binding regions relative to peak centre is plotted as a frequency distribution in (A) T-47D and (B) AB32 cells.
(PDF)

Figure S12 **PRE strength does not predict PR binding.** PRE motifs were classified in PR binding regions using the FIMO program in MEME [62]. The strength of the strongest candidate PRE, as determined by p value, in each binding region was plotted against peak height, as an indicator of PR binding strength. Estimated line of fit and Pearson correlation R^2^ value were estimated. Data are shown for (A) T-47D and (B) AB32 cells.
(PDF)

Figure S13 **FOXA1 transcript expression in cell lines.** FOXA1 transcript expression, measured on Illumina HT-12 arrays, was compared in breast cancer (T-47D, ZR-75-1) and transformed normal breast (AB9, AB32) cells. FOXA1 levels are expressed relative to the level in AB32 cells.
(PDF)

Figure S14 **FOXA1 binding at PR binding regions with or without predicted FOXA1 motifs.** The presence of FOXA1 motifs in PR binding regions was predicted using Homer software. PR binding regions predicted to bind FOXA1 and regions lacking FOXA1 binding motifs were separately analysed for actual FOXA1 binding enrichment. Average FOXA1 binding strength in T-47D from ChIP-seq is shown at PR binding regions containing FOXA1 motifs (blue line) and in PR binding regions that lacked any predicted FOXA1 motif (red line).
(PDF)

Figure S15 **PR expression in T-47D, AB32 and AB9 cells.** Proteins from whole cell extracts at the loading indicated were fractionated by denaturing 7.5% polyacrylamide-SDS gel electrophoresis and transferred to nitrocellulose membrane. PR protein bands were visualized as described in Materials and Methods.
(PDF)

Table S1 **Summary of gene functional annotation by chromosome in AB32.** Gene ontology term enrichment was determined for the subset of PR binding region-associated genes in AB32 cells on chromosomes 2, 8 and 11.
(PDF)

Table S2 **Validation of top PR binding regions.** PR binding to selected sites in T-47D and AB32 cells was confirmed by directed ChIP-PCR. Ten binding regions were selected for validation in T-47D and nine in AB32 cells.
(PDF)

Table S3 **Functional analysis of progestin regulated gene clusters lost, gained and conserved with expression of FOXA1.** Functional annotation clustering was performed for the groups of genes that lost, gained and conserved progestin regulation in AB32 cells after expression of FOXA1.
(PDF)
