# Supplementary figures and images for: Non-Overlapping Progesterone Receptor Cistromes Contribute to Cell-Specific Transcriptional Outcomes
Source: PLoS One. 2012 Apr 24;7(4):e35859. doi: 10.1371/journal.pone.0035859 (PMC3335806; doi:10.1371/journal.pone.0035859)

Figure S1

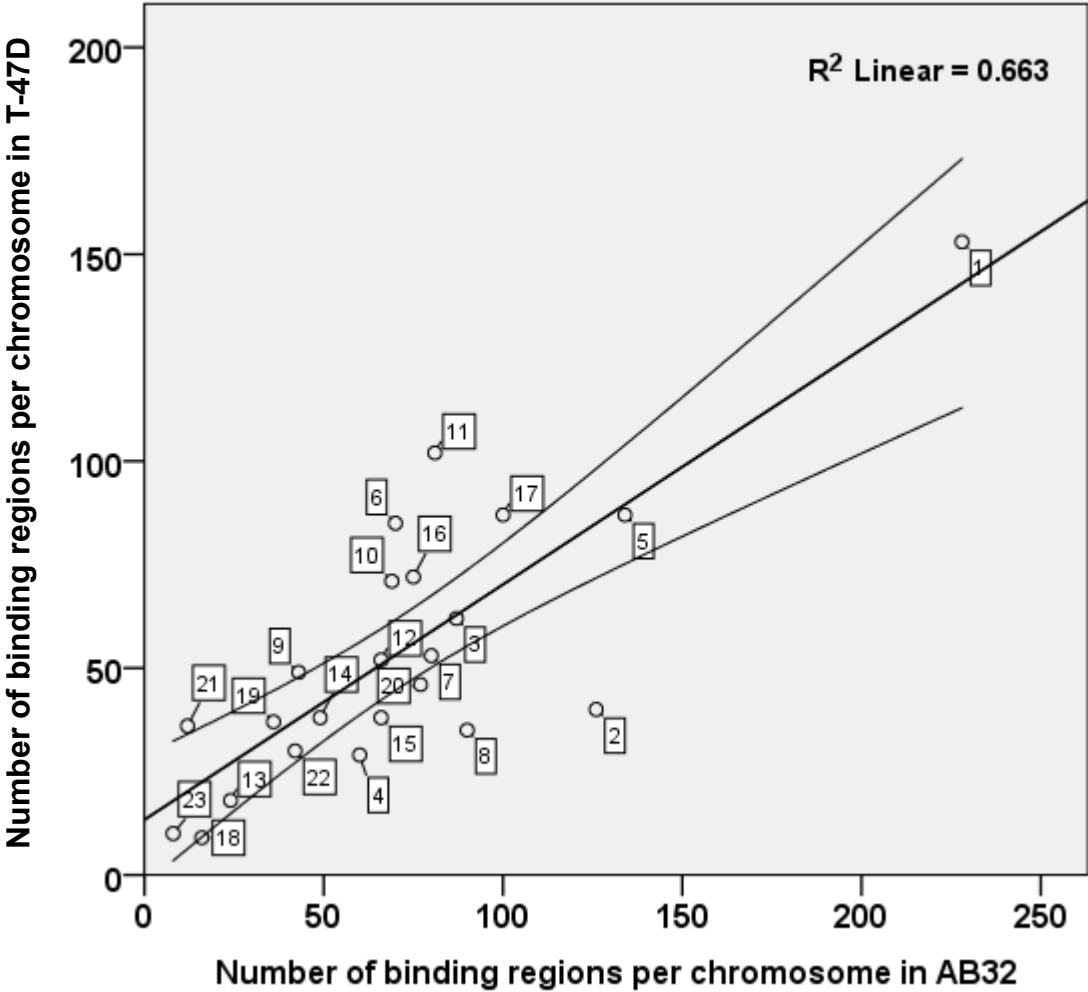

Supplement: Figure S1 — PR binding region to chromosome distribution in T-47D and AB32 cells. Total numbers of PR binding region per chromosome were compared by linear regression between T-47D and AB32 datasets. Line of fit and 95% confidence intervals are shown. Labels represent chromosome number. (PDF) [file pone.0035859.s001.pdf]

Figure S2

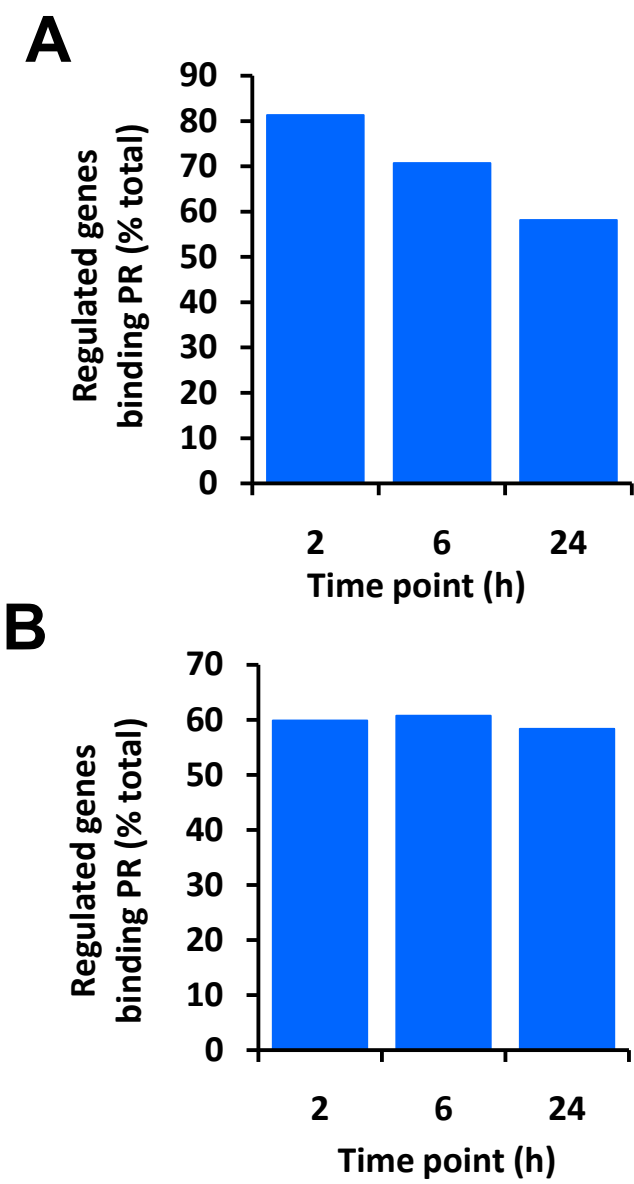

Supplement: Figure S2 — Relationship between PR binding and time of progestin regulation. The proportion of progestin regulated genes at 2, 6 or 24 h after treatment, which were associated with one or more PR binding regions was determined in (A) T-47D and (B) AB32 cells. (PDF) [file pone.0035859.s002.pdf]

Figure S3

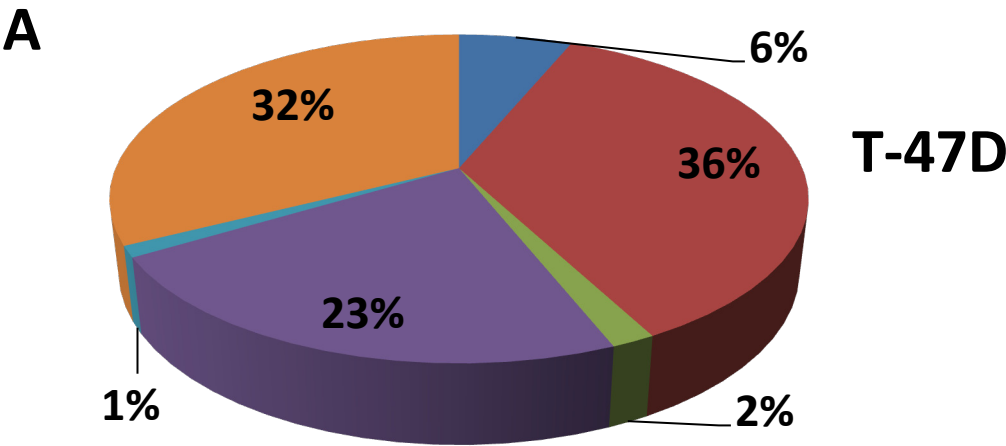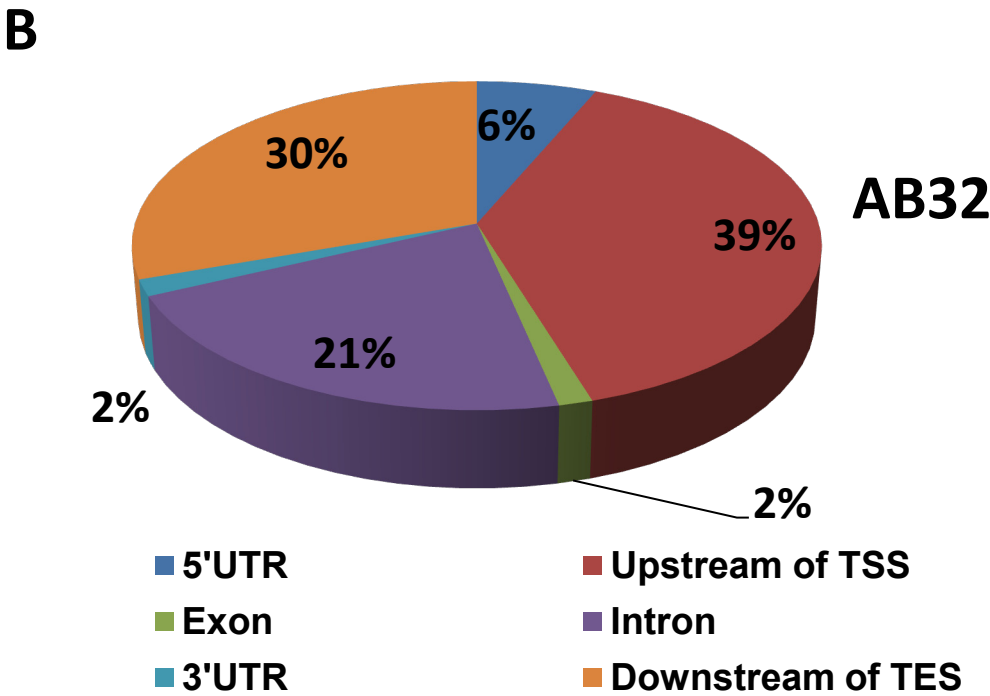

Supplement: Figure S3 — Location of PR binding regions. The distribution of all PR binding regions, with respect to the nearest gene, was determined using CisGenome v1.1 in (A) T-47D and (B) AB32 cells. (PDF) [file pone.0035859.s003.pdf]

Figure S4

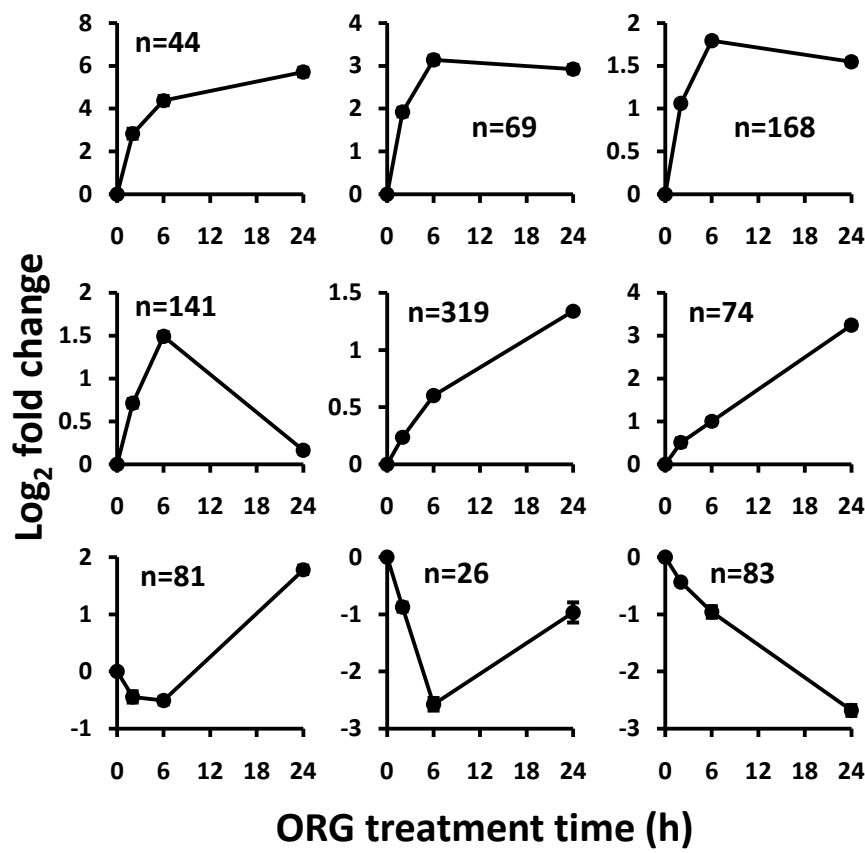

Supplement: Figure S4 — Patterns of transcriptional regulation in T-47D cells. Transcripts that were significantly differently expressed in 10 nM ORG2058 treated cells relative to vehicle at 2, 6 and 24 h after treatment were identified by gene expression profiling on Illumina HT-12 whole genome array. Self-organising map clustering was performed for all progestin regulated genes, using Gene Pattern. (PDF) [file pone.0035859.s004.pdf]

Figure S5

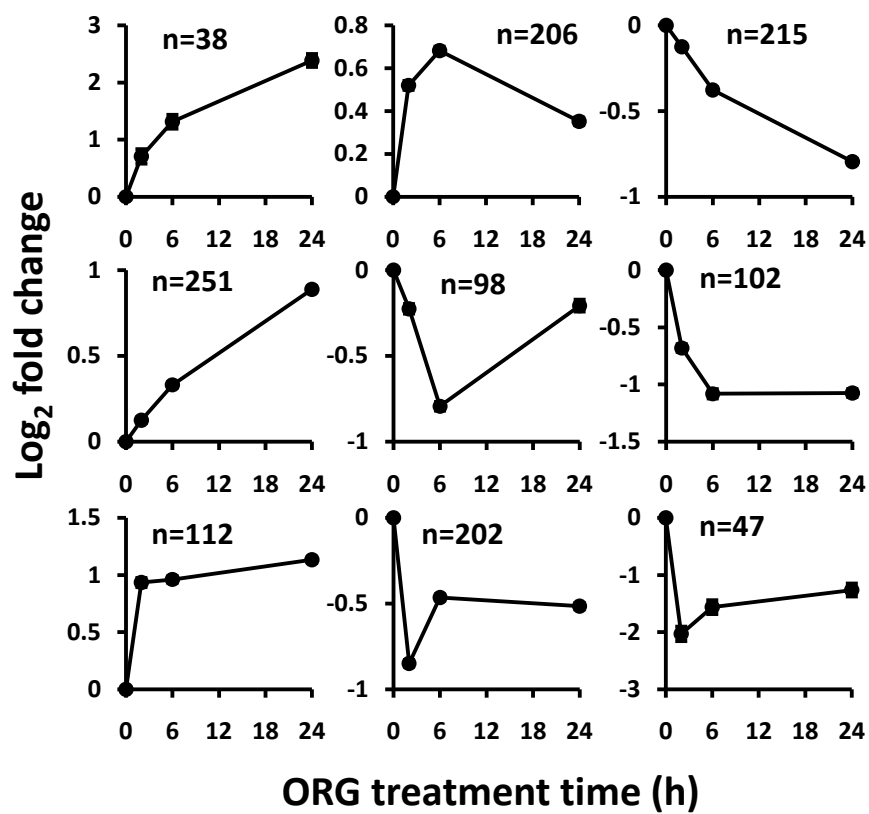

Supplement: Figure S5 — Patterns of transcriptional regulation in AB32 cells. Progestin regulated transcripts were identified in AB32 cells at 2, 6 and 24 h treatment with 10 nM ORG2058 by gene expression profiling. Patterns of transcriptional regulation over the 24 h time course were identified by self-organising map clustering using Gene Pattern. (PDF) [file pone.0035859.s005.pdf]

Figure S6

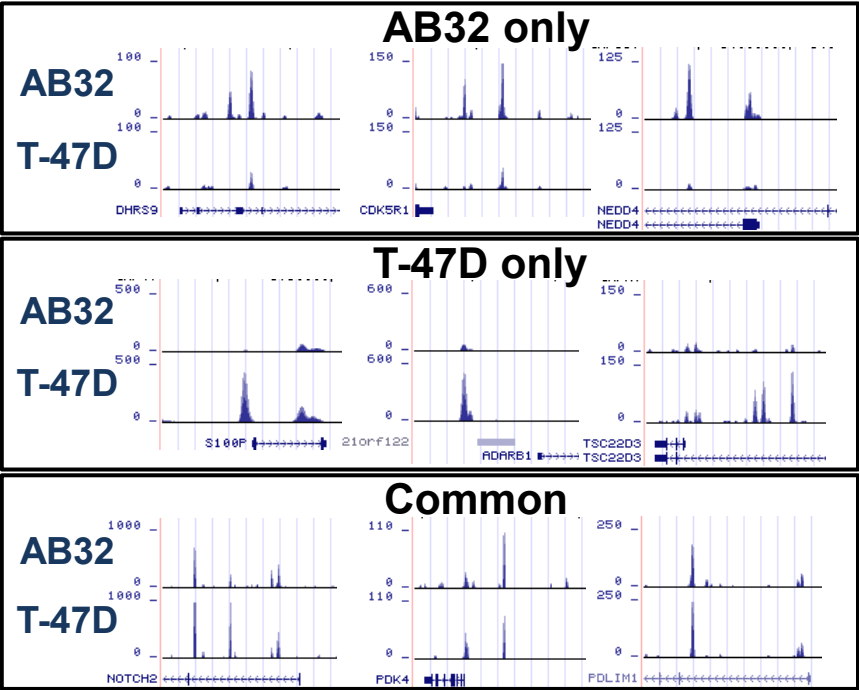

Supplement: Figure S6 — PR binding regions in T-47D and AB32 cells. Examples of PR binding regions that were unique to T-47D or AB32 cells or common to both lines are shown as custom tracks displayed in the UCSC genome browser. (PDF) [file pone.0035859.s006.pdf]

**A T-47D targets**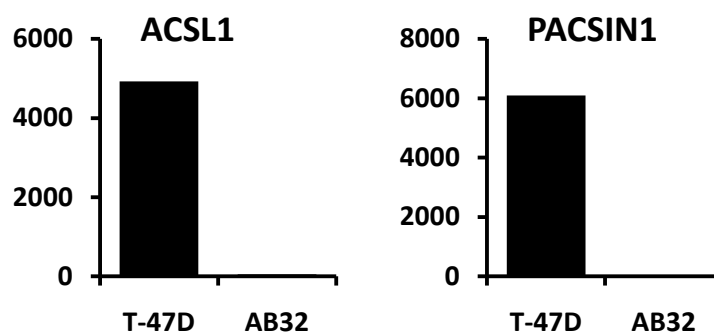**B AB32 targets**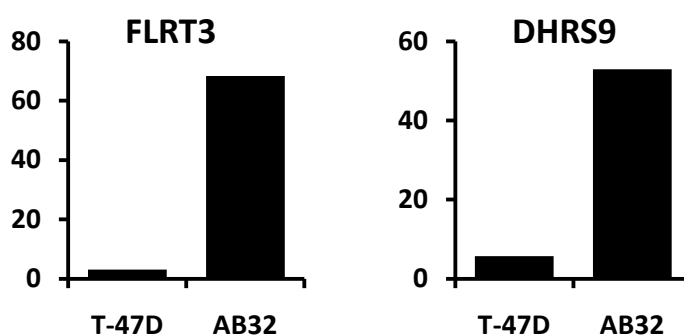**C Common targets**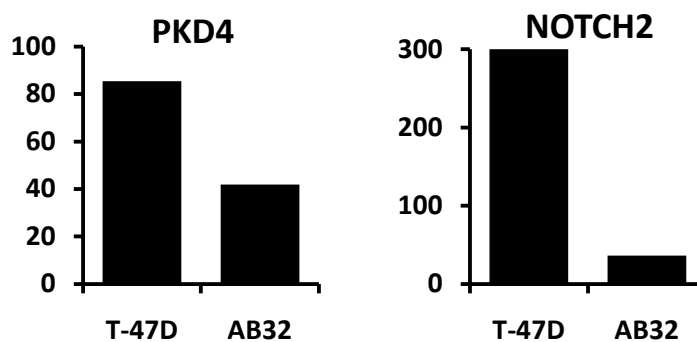

Supplement: Figure S7 — Validation of cell type-specific PR binding regions identified in ChIP-seq. PR binding regions identified in (A) T-47D, (B) AB32 or (C) both cell lines by ChIP-seq were validated by directed PR-ChIP, using binding region-specific primers and quantitative real-time PCR. Regions bound near ACSL1 and PACSIN1, which were regulated in T-47D but not AB32 produced marked enrichment of bound fragments in T-47D cells and not AB32. The converse was true with PR target regions identified in AB32 but not T-47D. FLRT3 and DHRS9, which are both transcriptional targets only in AB32, were strongly bound by PR in AB32 but showed a weak association in T-47D cells. PDK4 and Notch 2, which are progestin regulated in both cell lines, were bound by PR in both although the association was stronger in T-47D (85-fold vs42-fold binding enrichment of PDK-4 and 300-fold vs37-fold enrichment of Notch 2 binding in T-47D vs AB32). (PDF) [file pone.0035859.s007.pdf]

**Figure S8**

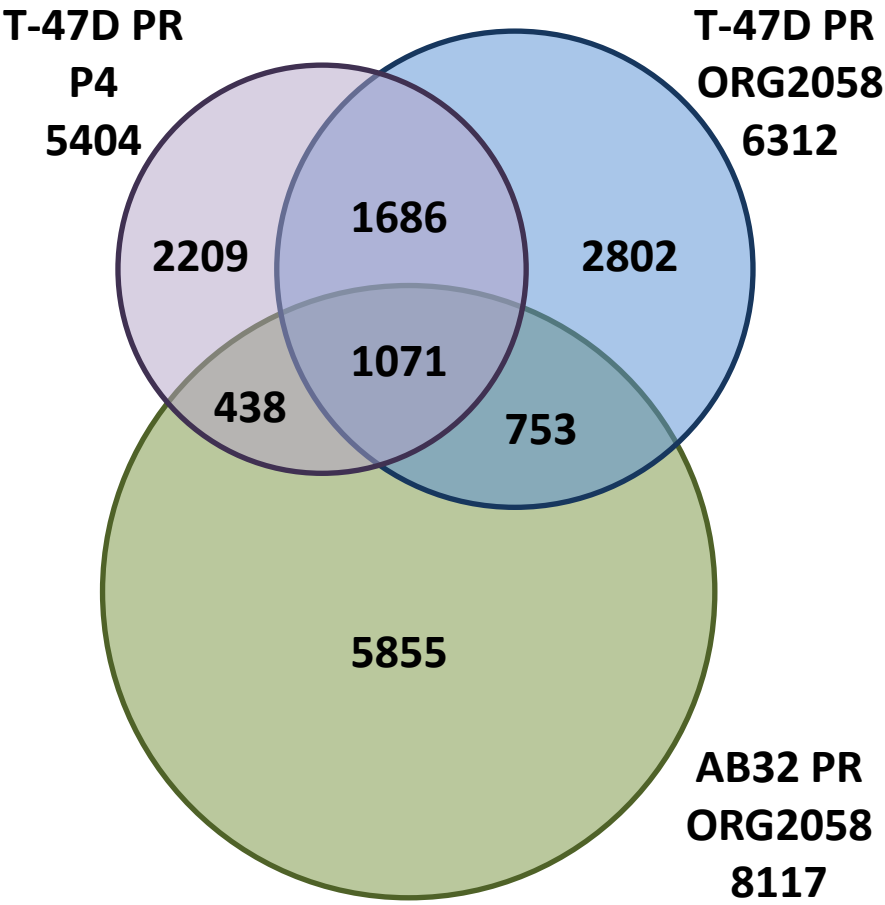

Supplement: Figure S8 — Overlap of PR binding regions in ORG2058-treated T-47D and AB32 with binding in T-47D after progesterone (P4) treatment. Our data are compared with progesterone-liganded PR binding in T-47D summarized in Tang et al [39] and available at http://cistrome.dfci.harvard.edu/NR_Cistrome/. (PDF) [file pone.0035859.s008.pdf]

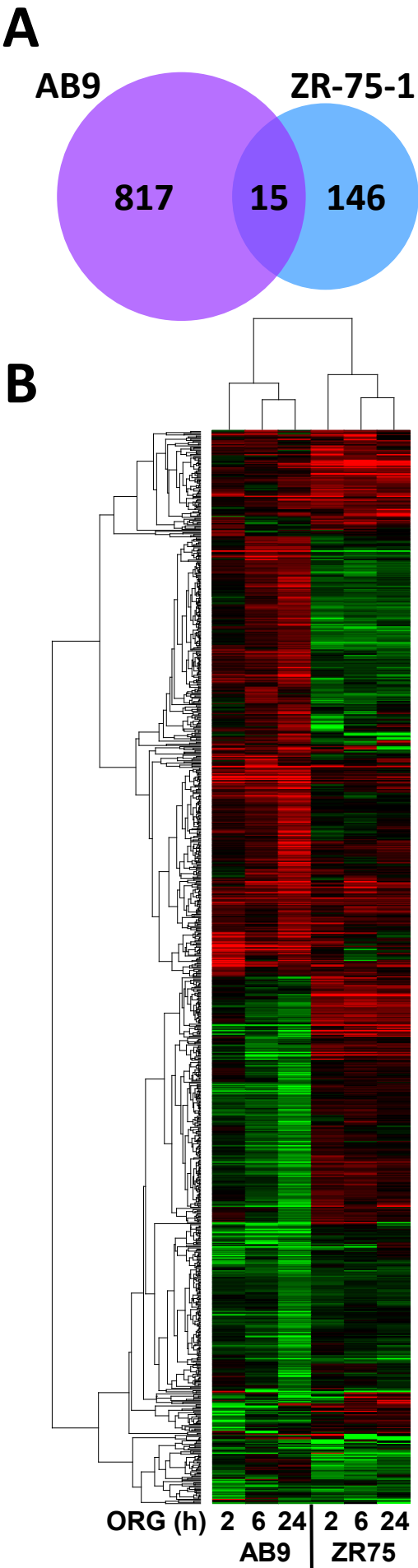

Supplement: Figure S9 — Progestin regulation of gene expression in additional breast cell lines. ZR-75-1 breast cancer cells and AB9 PR-positive transformed normal breast cells were treated for 2, 6 or 24 h with 10 nM ORG2058 or vehicle, then harvested and total RNA was isolated. Gene expression levels were estimated by Illumina HT-12 microarray. Data were analysed using Genome Studio software. Transcripts with levels that were significantly different in ORG compared to vehicle-treated cells (diff p value<0.01) and had a fold change of 1.5 or more were considered progestin regulated. (A) Numbers of progestin regulated transcripts in ZR-75-1 or AB9 cells or both. (B) Unsupervised average linkage hierarchical cluster analysis of arrays (Pearson correlation) and gene expression fold change (uncentred correlation) was performed on the subset of transcripts that were progestin regulated in one or both cell lines, using Gene Pattern. Red - increased expression, green - decreased expression with ORG, relative to vehicle. (PDF) [file pone.0035859.s009.pdf]

A

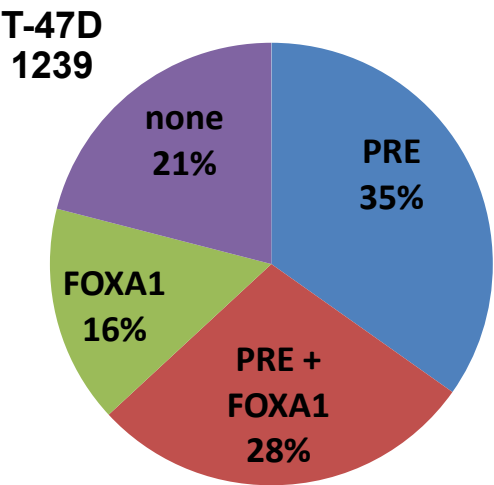

B

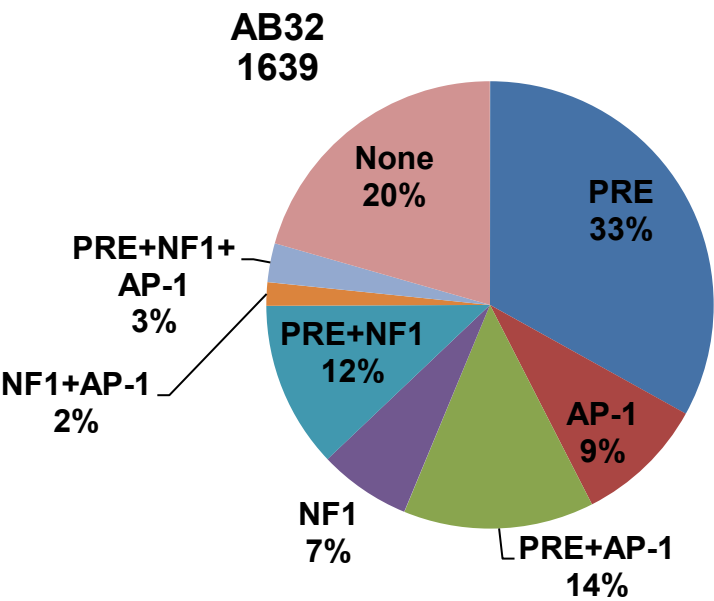

Supplement: Figure S10 — PRE and cofactor motif enrichment in regulation-associated binding sites in T-47D and AB32 cells. The relative proportions of regulation-associated PR binding regions containing PREs with or without one or more of the top enriched transcriptional cofactor binding motifs are shown. (A) T-47D and (B) AB32 motif distribution. (PDF) [file pone.0035859.s010.pdf]

A

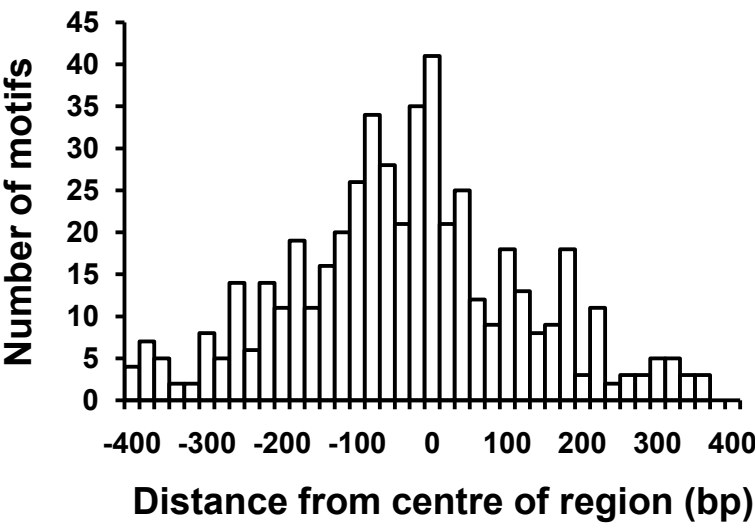

B

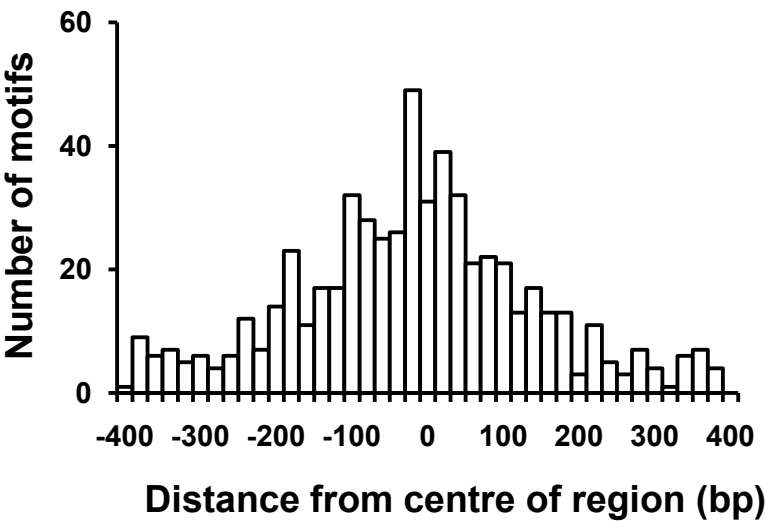

Supplement: Figure S11 — Distribution of PRE position in PR binding regions in T-47D and AB32 cells. The positions of PRE motifs in PR binding regions relative to peak centre is plotted as a frequency distribution in (A) T-47D and (B) AB32 cells. (PDF) [file pone.0035859.s011.pdf]

A

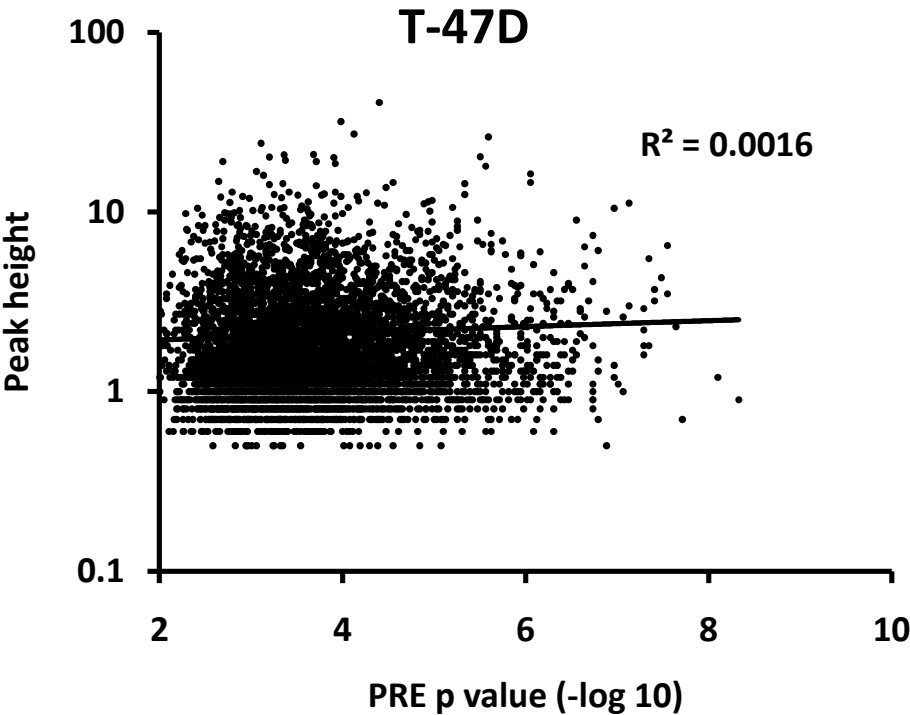

B

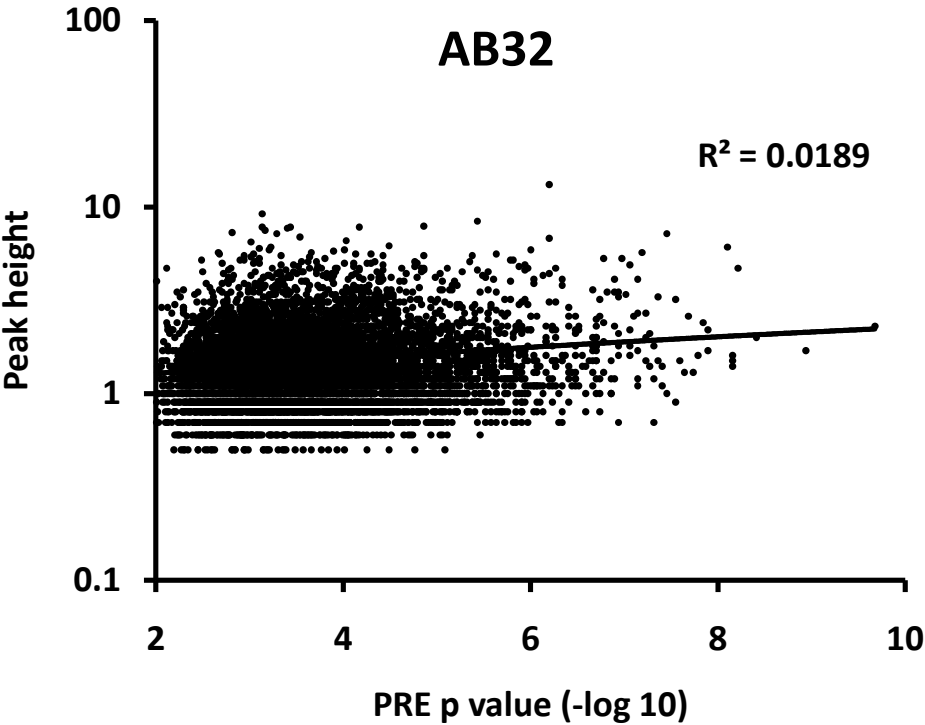

Supplement: Figure S12 — PRE strength does not predict PR binding. PRE motifs were classified in PR binding regions using the FIMO program in MEME [62]. The strength of the strongest candidate PRE, as determined by p value, in each binding region was plotted against peak height, as an indicator of PR binding strength. Estimated line of fit and Pearson correlation R2 value were estimated. Data are shown for (A) T-47D and (B) AB32 cells. (PDF) [file pone.0035859.s012.pdf]

**Figure S13**

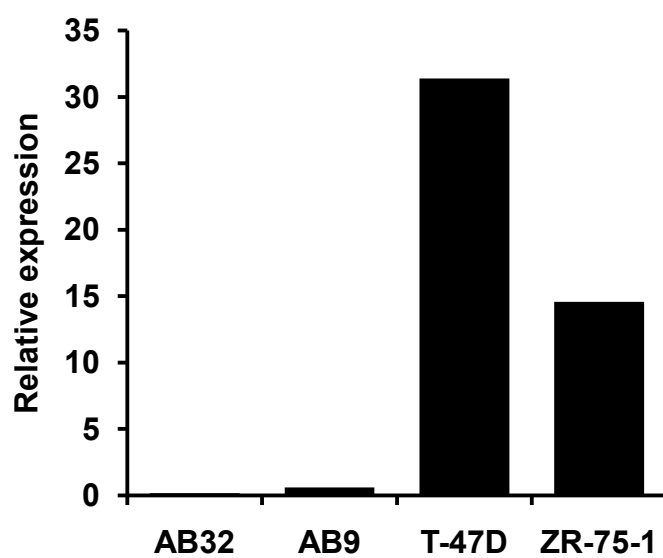

Supplement: Figure S13 — FOXA1 transcript expression in cell lines. FOXA1 transcript expression, measured on Illumina HT-12 arrays, was compared in breast cancer (T-47D, ZR-75-1) and transformed normal breast (AB9, AB32) cells. FOXA1 levels are expressed relative to the level in AB32 cells. (PDF) [file pone.0035859.s013.pdf]

**Figure S14**

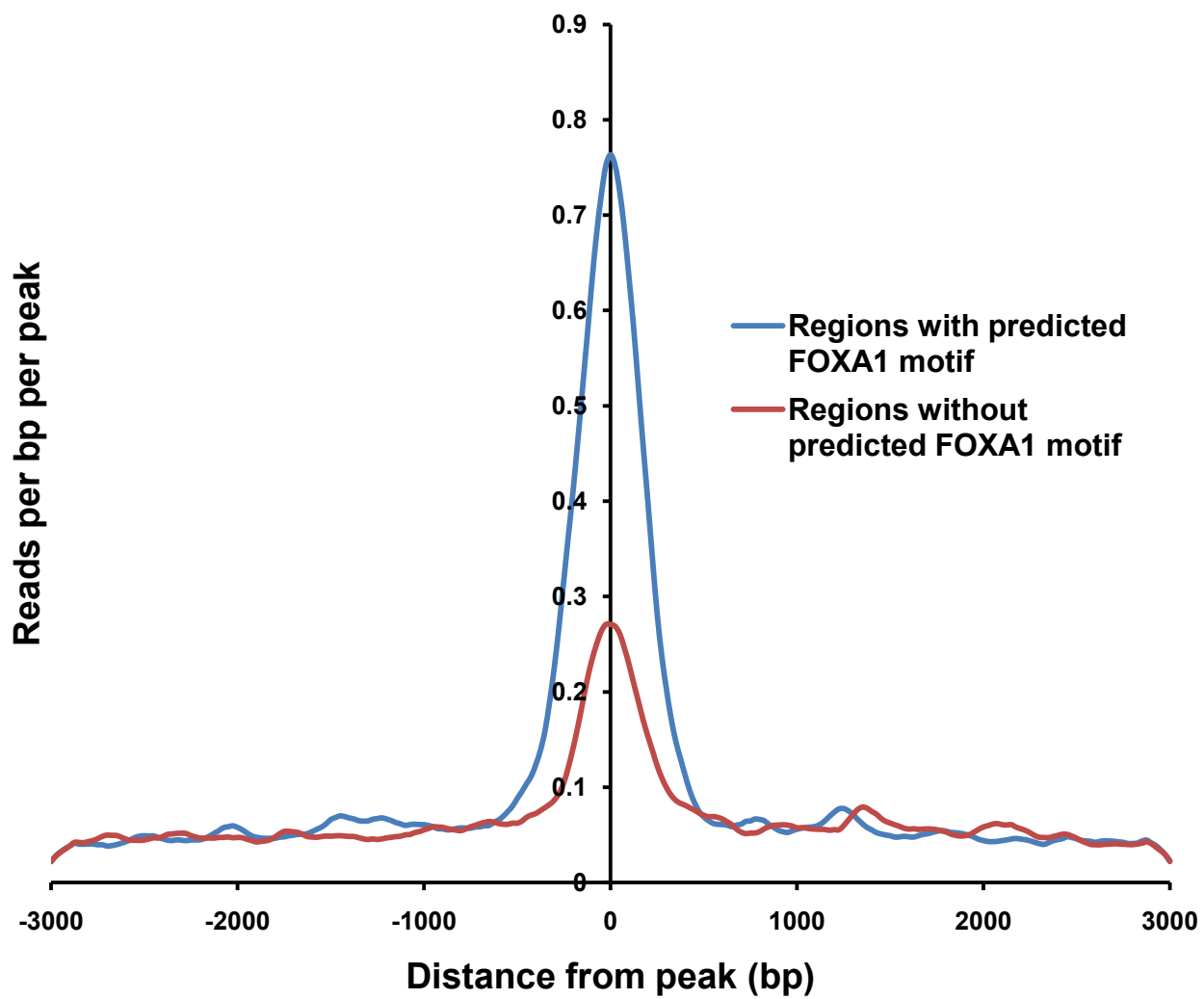

Supplement: Figure S14 — FOXA1 binding at PR binding regions with or without predicted FOXA1 motifs. The presence of FOXA1 motifs in PR binding regions was predicted using Homer software. PR binding regions predicted to bind FOXA1 and regions lacking FOXA1 binding motifs were separately analysed for actual FOXA1 binding enrichment. Average FOXA1 binding strength in T-47D from ChIP-seq is shown at PR binding regions containing FOXA1 motifs (blue line) and in PR binding regions that lacked any predicted FOXA1 motif (red line). (PDF) [file pone.0035859.s014.pdf]

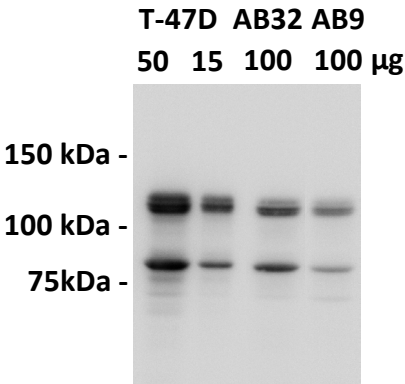

Supplement: Figure S15 — PR expression in T-47D, AB32 and AB9 cells. Proteins from whole cell extracts at the loading indicated were fractionated by denaturing 7.5% polyacrylamide-SDS gel electrophoresis and transferred to nitrocellulose membrane. PR protein bands were visualized as described in Materials and Methods. (PDF) [file pone.0035859.s015.pdf]
