# Supplementary material for: Non-Overlapping Progesterone Receptor Cistromes Contribute to Cell-Specific Transcriptional Outcomes
Source: PLoS One. 2012 Apr 24;7(4):e35859. doi: 10.1371/journal.pone.0035859 (PMC3335806; doi:10.1371/journal.pone.0035859)
Supplement: Table S1 — Summary of gene functional annotation by chromosome in AB32. Gene ontology term enrichment was determined for the subset of PR binding region-associated genes in AB32 cells on chromosomes 2, 8 and 11. (PDF) [file pone.0035859.s016.pdf]

Table S1: Summary of gene functional annotation by chromosome in AB32

Gene ontology term enrichment was determined for the subset of PR binding region-associated genes in AB32 cells on chromosomes 2, 8 and 11.

**Chr 2 (58 IDs)**<br>**Annotation Cluster**	**Enrichment Score**	**Function**
1	2.75	Metabolism
2	1.61	GTPase activity
3	0.91	Metabolism

**Chr 8 (25 IDs)**<br>**Annotation Cluster**	**Enrichment Score**	**Function**
1	1.62	Apoptosis
4	0.72	Catabolism
6	0.55	Transcriptional regulation
7	0.52	Apoptosis
8	0.39	Nucleotide binding
11	0.11	Metal ion binding

**Chr 11 (39 IDs)**<br>**Annotation Cluster**	**Enrichment Score**	**Function**
1	1.57	Metabolism
3	0.73	DNA repair
4	0.72	Protein complex assembly
5	0.70	Transcriptional regulation
6	0.63	Phosphorylation
7	0.51	Transcriptional regulation
8	0.37	Apoptosis
