# Supplementary material for: Non-Overlapping Progesterone Receptor Cistromes Contribute to Cell-Specific Transcriptional Outcomes
Source: PLoS One. 2012 Apr 24;7(4):e35859. doi: 10.1371/journal.pone.0035859 (PMC3335806; doi:10.1371/journal.pone.0035859)
Supplement: Table S2 — Validation of top PR binding regions. PR binding to selected sites in T-47D and AB32 cells was confirmed by directed ChIP-PCR. Ten binding regions were selected for validation in T-47D and nine in AB32 cells. (PDF) [file pone.0035859.s017.pdf]

Table S2 - Validation of top PR binding regions

T-47D

Region ID	Region position			Peak Height	PRE p-value	PRE score	Validation: Fold enrichment
	chromosome	start	end				
T47D4122	chr3	184316710	184317034	40.7	3.96E-05	++	3.97
T47D6084	chr9	122679651	122680502	26.2	2.55E-06	+++	5.68
T47D6046	chr9	111991161	111991613	24.1	7.76E-04	++	46.53
T47D1380	chr11	128699096	128700220	20.9	2.07E-04	++	4.08
T47D2524	chr16	84310655	84311589	20.8	4.35E-04	++	1.29
T47D283	chr1	120376407	120376763	20.2	6.29E-04	++	7.14
T47D3464	chr20	44776235	44776939	20.1	1.24E-04	++	12.27
T47D5837	chr8	102519793	102520361	19.1	2.03E-03	+	13.06
T47D3514	chr20	57834740	57835410	14.6	8.84E-07	+++	4.34
T47D4818	chr5	148845545	148845884	12.9	1.20E-04	++	13.29

AB32

Region ID	Region position			Peak Height	PRE p-value	PRE score	Validation: Fold enrichment
	chromosome	start	end				
AB32399	chr1	143957617	143957902	13.2	6.32E-07	+++	6.49
AB326156	chr5	141702092	141702799	9.2	7.33E-04	++	2.01
AB326110	chr5	134596597	134597211	8.4	3.69E-06	+++	16.08
AB324311	chr2	223025077	223026178	7.9	1.38E-05	++	9.92
AB324058	chr2	114040782	114041064	7.8	3.66E-04	++	1.24
AB32833	chr1	227827184	227827908	7.8	7.33E-04	++	6.28
AB323972	chr2	91137645	91137989	7.8	6.73E-05	++	2.86
AB324554	chr20	33358266	33358837	7.2	3.51E-08	+++	0.91
AB32810	chr1	223989877	223991355	7.2	5.12E-04	++	2.21
