# Supplementary material for: Non-Overlapping Progesterone Receptor Cistromes Contribute to Cell-Specific Transcriptional Outcomes
Source: PLoS One. 2012 Apr 24;7(4):e35859. doi: 10.1371/journal.pone.0035859 (PMC3335806; doi:10.1371/journal.pone.0035859)
Supplement: Table S3 — Functional analysis of progestin regulated gene clusters lost, gained and conserved with expression of FOXA1. Functional annotation clustering was performed for the groups of genes that lost, gained and conserved progestin regulation in AB32 cells after expression of FOXA1. (PDF) [file pone.0035859.s018.pdf]

Table S3: Functional analysis of progestin regulated gene clusters lost, gained and conserved with expression of FOXA1.

Functional group	Enrichment Score
Lost	
Apoptosis	6.68
Steroid biosynthesis	2.90
Regulation of protein kinase activity	2.41
Protein transport	2.17
Gained	
Blood vessel morphogenesis	3.28
Cell motility	2.03
Conserved	
Mitotic cell cycle	4.37
Wound healing	3.55
Apoptosis	2.40
